# A Novel Approach Identifying Hybrid Sterility QTL on the Autosomes of *Drosophila simulans* and *D. mauritiana*

**DOI:** 10.1371/journal.pone.0073325

**Published:** 2013-09-05

**Authors:** Christopher T. D. Dickman, Amanda J. Moehring

**Affiliations:** Department of Biology, Western University, London, Ontario, Canada; The University of Queensland, St. Lucia, Australia

## Abstract

When species interbreed, the hybrid offspring that are produced are often sterile. If only one hybrid sex is sterile, it is almost always the heterogametic (XY or ZW) sex. Taking this trend into account, the predominant model used to explain the genetic basis of F_1_ sterility involves a deleterious interaction between recessive sex-linked loci from one species and dominant autosomal loci from the other species. This model is difficult to evaluate, however, as only a handful of loci influencing interspecies hybrid sterility have been identified, and their autosomal genetic interactors have remained elusive. One hindrance to their identification has been the overwhelming effect of the sex chromosome in mapping studies, which could ‘mask’ the ability to accurately map autosomal factors. Here, we use a novel approach employing attached-X chromosomes to create reciprocal backcross interspecies hybrid males that have a non-recombinant sex chromosome and recombinant autosomes. The heritable variation in phenotype is thus solely caused by differences in the autosomes, thereby allowing us to accurately identify the number and location of autosomal sterility loci. In one direction of backcross, all males were sterile, indicating that sterility could be entirely induced by the sex chromosome complement in these males. In the other direction, we identified nine quantitative trait loci that account for a surprisingly large amount (56%) of the autosome-induced phenotypic variance in sterility, with a large contribution of autosome-autosome epistatic interactions. These loci are capable of acting dominantly, and thus could contribute to F_1_ hybrid sterility.

## Introduction

Reproductive isolation occurs when there is a barrier that prevents two species from producing fit hybrid offspring. An interesting, and well-documented, phenomenon of hybrid dysfunction is that it is more likely to affect the heterogametic (XY or ZW) sex, a trend known as ‘Haldane’s Rule’ [1]. While the faster-male evolution theory explains Haldane’s Rule in species where the male is the heterogametic sex [2], [3], it does not explain why the trend extends to species in which the female is the heterogametic sex. As such, one of the predominant models for explaining this trend is a combination of the Dobzhansky-Muller (D-M) model and the dominance model, whereby dysfunction in the interspecies F_1_ is caused by a deleterious interaction between a recessive sex-linked factor from one species and a dominant autosomal factor from the other species [4]–[7]. The dominance of the autosomal factor explains how it affects an interspecies F_1_; the recessive sex-linked factor is masked in homogametic individuals and unmasked in heterogametic individuals, thus explaining the appearance of sterility in heterogametic individuals, and Haldane’s Rule.

To date only four genes affecting interspecies hybrid sterility in animals have been characterized [8]–[11]. In addition, several studies have mapped genomic regions containing autosomal sterility loci using introgressions (e.g., [12]–[15]). However, most of these studies do not address the genetic conditions that underlie F_1_ hybrid sterility as they utilize a genetic background in which the autosomal factors can act recessively to induce sterility. Indeed, an abundance of recessive autosomal loci are typically found in introgression studies of hybrid sterility, while dominant loci are not identified. Thus, of the identified loci, only *Overdrive* acts in a manner consistent with the predominant theoretical model, and the individual interactor loci have not yet been identified [16].

**Figure 1:**
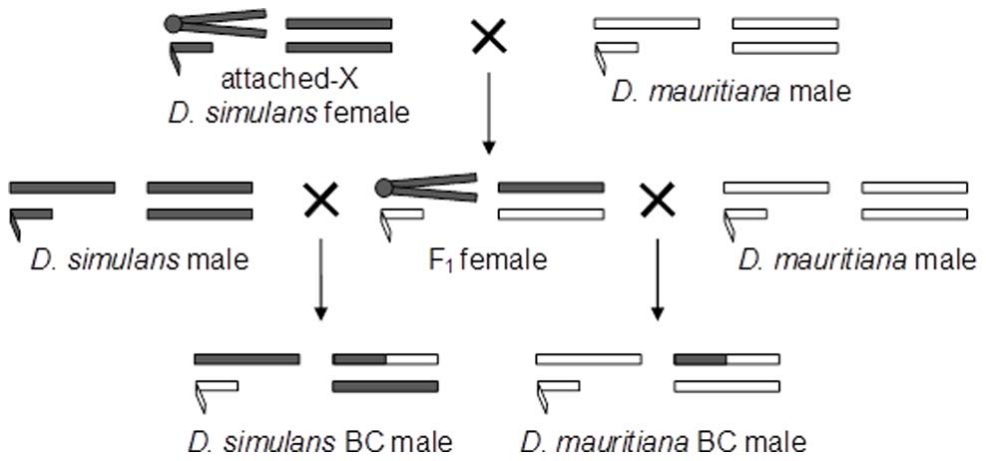
Crossing scheme used to obtain backcross males. This diagram represents all homologous pairs of autosomes as a pair of bars on the right for each individual. Sex chromosomes are on the left for each individual, with small hooked bars representing Y chromosomes, longer bars representing X chromosomes, and two joined bars representing the attached-X chromosome. Grey denotes *D. simulans* genetic material and white *D. mauritiana* material. Note that attached-X females also carry a Y chromosome, but remain female due to the mechanism of sex determination in *Drosophila*.

One approach that has been utilized in a variety of species for identifying dominant autosomal loci influencing hybrid sterility has been quantitative trait locus (QTL) mapping. However, there is a disproportionate contribution of the X chromosome to hybrid sterility (e.g., [17]–[19]), and the autosomal component of these genetic maps can potentially be masked by the excessively large effect of the sex chromosome (e.g., [20], [21]). Here, we use the recently-diverged species pair *Drosophila simulans* and *D. mauritiana*; when these species are crossed, sterile F_1_ males and fertile F_1_ females are produced. While several studies have fine-mapped the location of X-linked factors affecting sterility (e.g., [22], [23]), these loci do not act in a manner that would produce F_1_ hybrid sterility, and the location of autosomal loci contributing to sterility has largely remained unexplored. Surprisingly, although extensively studied as a genetic model for hybrid sterility, a QTL map of sterility has never been performed in this species pair. We therefore perform QTL mapping for sterility in this pair, but with a particular focus on autosomal dominant loci. To allow for detection of sterility factors in the two species’ genetic backgrounds, we created two mapping populations by backcrossing the fertile F_1_ females to both parental species. To bypass the potentially overwhelming X-chromosome effect and improve the resolution of the autosomal genetic map, we implement a creative use of flies with an attached-X genetic construct, allowing us to generate offspring with non-recombinant sex chromosomes and recombinant autosomes (Figure 1). This enabled us to create a refined map of autosomal loci contributing to hybrid sterility by eliminating any confounding effects of variable sex chromosomes. It also enhanced our ability to detect complex epistatic incompatibilities that involve an autosomal interaction with multiple X-linked loci. The unique genetic complement of the mapping population has thus allowed us to more accurately identify the number, location, effect size, and epistatic interactions of dominant autosomal hybrid sterility loci.

## Materials and Methods

### Stocks and Crosses

All flies were maintained on a 14∶10 hour light:dark cycle at 24°C on standard cornmeal/molasses/agar medium. *D. mauritiana* synthetic (SYN; [24]), *D. simulans* Florida City (FC; [24]), and *D. simulans* attached-X (*C(1)RM w*/*lz^S^*; provided by D. Presgraves) were used. As sex determination in *Drosophila* is dosage dependent, all attached-X individuals are female, even though they also contain a Y chromosome. Virgin attached-X *D. simulans* females were aged five days, and then crossed with 1–6 day-old *D. mauritiana* males. F_1_ females were immediately backcrossed to males of one of the parental species: *D. simulans* FC or *D. mauritiana* SYN (Figure 1). Backcross males from both crosses were collected within 10 hours of eclosion and aged for 3 to 5 days to ensure reproductive maturity prior to scoring the sperm phenotype.

### Sperm Motility Assays

Sperm motility was assayed as a proxy for male fertility. Although it is possible for a male with motile sperm to be sterile, this method has been shown to account for most cases of infertility [25]. Males were placed in Biggers–Whitten–Whittingham buffer [26] and the testes were removed. The body of the fly, except for the testes, was frozen for later DNA analysis. The testes were gently burst open underneath a glass coverslip and observed under a light microscope using phase contrast. It is not possible to accurately count the number of motile sperm using this method, and so each individual was scored for the presence of sperm and whether or not sperm was motile. 266 *D. simulans* backcross and 760 *D. mauritiana* males were dissected. As a control, 10 three-day-old males each of *D. simulans* FC and *D. mauritiana* SYN were also assayed for sperm motility.

### QTL Analysis

Genotyping was completed using microsatellite analysis for 20 markers throughout the second and third chromosomes (Table S1). The primers were initially tested on 5 *D. simulans* and 5 *D. mauritiana* flies to ensure that the markers were divergent between the two species, but not polymorphic within each species. The markers on the second chromosome were amplified individually using PCR and run on a 3% agarose gel. Markers on the third chromosome were amplified using fluorescently-labeled primers in a multiplex PCR reaction and the samples were analyzed using capillary electrophoresis at the Michael Smith Laboratories Nucleic Acid Protein Service Unit (BC, Canada). An X chromosome marker was used to confirm there was no contamination of the stocks or separation of the attached-X chromosome, which would cause the backcross males to receive an X chromosome from the alternate species.

QTL mapping was performed in three different ways: 1) using sperm motility as a binary trait: presence of motile sperm *vs.* absence of motile sperm (which includes both non-motile sperm and the absence of sperm), 2) using all three categories: presence of motile sperm *vs.* presence of non-motile sperm *vs.* absence of sperm, and 3) using sperm presence as a binary trait: presence of sperm (motile or non-motile) *vs.* absence of sperm. QTL were mapped using Windows QTL Cartographer V.2.5 [27]. Composite interval mapping (CIM; [28]) was performed using a window size of 10 in a forward-backward regression. In CIM, the WinQTLCart software calculates a logarithm of the odds ratio (LOD) score using log_10_ of the formula 2log(L_0_/L_1_), where L_0_ is the likelihood that there is no QTL within a given interval between two markers (the null hypothesis) and L_1_ is the likelihood of the alternate hypothesis that there is a QTL within an interval. The higher the LOD value, the higher the likelihood that there is a gene affecting the trait of interest within that region. One thousand permutations were performed [29] for each data set to determine the significance threshold of *p*≤0.05. The effect size (*R* ^2^) of each QTL peak was estimated by calculating the difference between the values of the phenotype for heterozygotes and homozygotes under the peak LOD value for each QTL, and then scaling for the standard deviation of the phenotypic value. This value was multiplied by 100 to calculate the value we call %V_P_. The total effect size (T*R* ^2^) is calculated the same way, but includes the effect of background cofactors; this value was multiplied by 100 to calculate the value we call %TV_P_. The QTL position’s 95% confidence interval was determined by a 1.5-LOD interval surrounding the highest LOD for each QTL [30]. While our data is categorical, and thus violates the assumption of normality for CIM mapping, previous work [31] has shown that CIM is very robust to departures from normality.

We refined the QTL positions and tested for epistasis among the QTL using Multiple Interval Mapping (MIM; [32]. The QTL positions were refined using the Refine Model in MIM. The first scan for epistasis using MIM was between the QTLs identified with CIM (QTL-QTL). As our results indicated that a portion of the phenotypic variance remained unexplained, suggesting that there may be epistatic effects beyond those between QTLs (e.g. between a QTL and a non-significant region of the genome), we also used MIM to perform a 1D scan for epistasis between each QTL and the remainder of the genome (QTL-other). For all epistasis tests, we used a walk speed of 1 cM, a window size of 10, and a significance threshold of 0.10. The larger significance threshold (*vs.* the traditional 0.05) was used since it has been shown that this increased value is more likely to capture significant effects but does not compromise the number of false positives [33].

## Results

**Figure 2:**
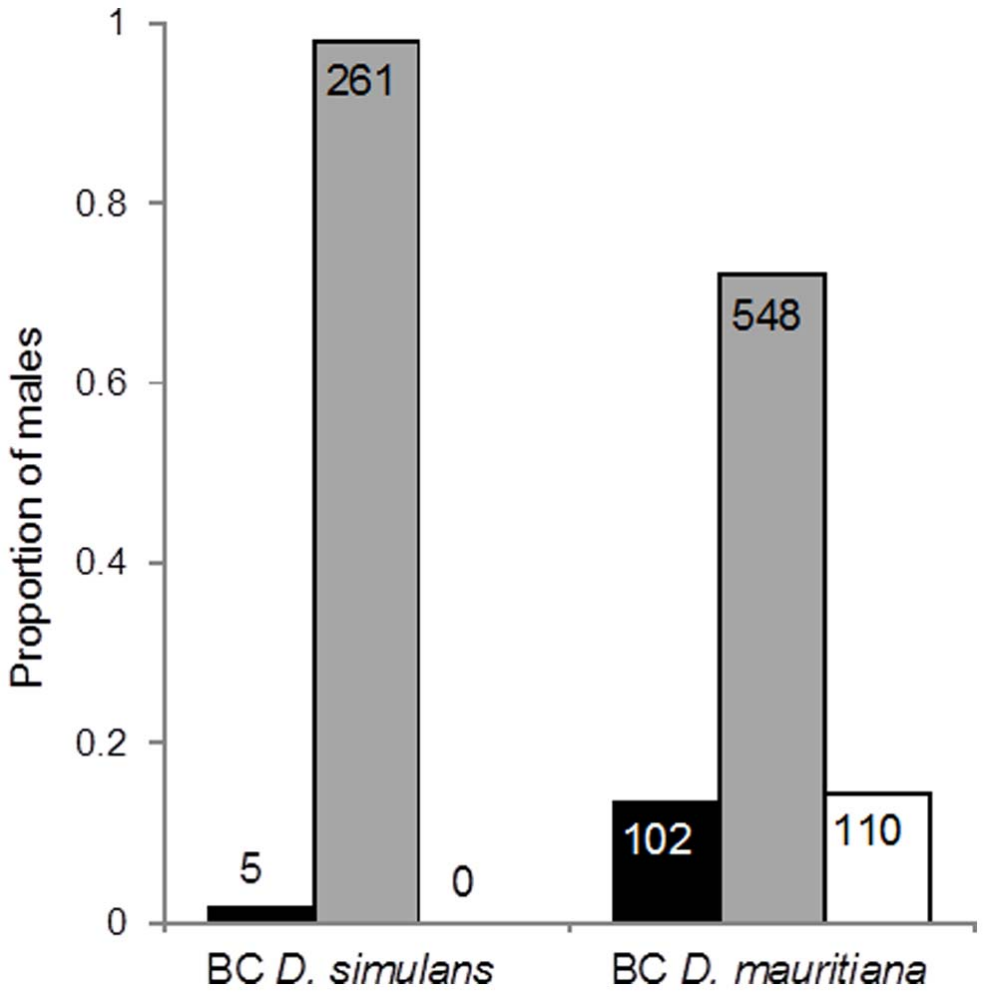
Distribution of sperm scores in backcross (BC) males. Proportion of BC *D. simulans* (on left) and *D mauritiana* (on right) males that had no sperm present (black bars), sperm present but non-motile (grey bars), or motile sperm (white bars). The number on each bar represents the number of males in each category.

Sperm presence and motility were assayed in backcross males who inherited a non-recombinant X chromosome (Figure 1); these males were then genotyped for QTL mapping of their autosomes. None of the males resulting from a backcross to *D. simulans* had motile sperm (N = 266), while approximately 15% of *D. mauritiana* backcross males (111 out of 760) had motile sperm (Figure 2). As a procedural control, pure species males were tested for sperm motility as well. All *D. simulans* males (n = 10) and nine out of ten *D. mauritiana* males had motile sperm, similar to the results of previous studies [34].

**Figure 3:**
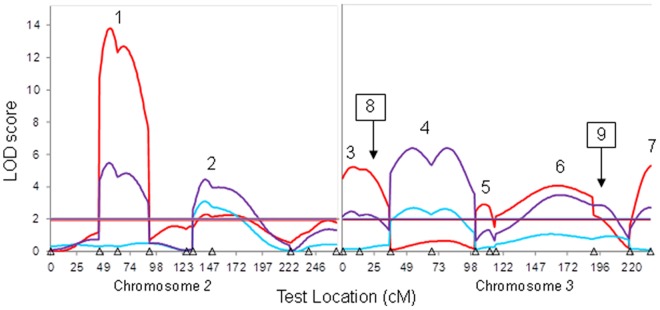
Composite interval map of *D. mauritiana* backcross male fertility. The second chromosome is on the left and the third chromosome on the right. Red represents a comparison of individuals based on presence or absence of motile sperm; purple is the same, but also includes information on sperm presence or absence; blue represents the analysis based solely on presence or absence of sperm. The correspondingly-colored horizontal lines show the significance thresholds for each trait; note that the thresholds for each trait are close in value and are overlapping in the figure. Triangles along the x-axis represent the locations of molecular markers used in genotyping. The number over each peak represents the QTL number for QTLs with main effects (Table 1); boxed numbers with an arrow represent QTLs that are only present when epistatic interactions are considered (Table 2).

As *D. simulans* backcross males were all sterile, it was not possible to perform QTL mapping to examine fertility, and so only the *D. mauritiana* backcross males were genotyped and analyzed. One set of these DNA samples were degraded, and thus 672 samples were genotyped (Table S2). The first QTL map comparison separated fertility scores into two categories: individuals with motile sperm and individuals without motile sperm, independent of the presence/absence of sperm in the latter category. Six QTL were identified as contributing to the presence or absence of motile sperm (red line, Figure 3; Table 1). Two of these QTL have an epistatic interaction with each other, and three have an epistatic interaction with a locus at 17 cM on the third chromosome (Table 2), indicating that QTL #3 is actually likely to be two closely-linked QTLs. As a whole, these QTL and their interactions account for 55.8% of the variance in the sterility phenotype (%V_P_; Tables 1, 2). The second comparison was similar to the first but included the presence of non-motile sperm as an intermediate trait between sperm absence and motile sperm. This map is similar to that produced from the first comparison, with the addition of QTL #4 in the middle of the second chromosome (purple line, Figure 3; Table 1). Two of these QTL have an epistatic interaction with each other, and one has an epistatic interaction with a locus at 197 cM on the third chromosome (Table 2), identifying an additional QTL that is within this region. The QTL and their epistatic interactions when examining both sperm presence and motility together account for 29.2% of the phenotypic variance (%V_P_; Tables 1, 2). The third comparison mapped QTL based on the presence or absence of sperm, regardless of motility. This yielded only two significant QTLs (blue line, Figure 3; Table 1), which contribute 5.5% of the phenotypic variance; no epistatic interactions were detected when the data were partitioned by this phenotype. Combining these results, seven QTL located throughout the autosomes were identified as main-effect QTLs, plus an additional two QTLs were identified as contributing to the trait when epistatic interactions were taken into consideration (Figure 3; Tables 1, 2). The additive effects of all of the main-effect QTLs for all traits measured were all positive values, confirming that sterility was due to the presence of *D. simulans* genome in the *D. mauritiana* genetic background, as expected. To further confirm this, genotypes for each marker closest to the QTL peaks were sorted by phenotype; the presence of *D. mauritiana* at each QTL location was correlated with a decrease in fertility (Table S3), indicating that these loci are acting dominantly.

**Table 1:** Hybrid sterility QTL locations and their effects.

Comparison	QTL #^1^	Chr.	cM^2^	Refined cM^2^	Range (cM)^3^	Max. LOD^4^	Additive Effect^5^	%V_P_ 6	%TV_P_ 7
Sperm motile *vs.* non-motile (present and absent)	1	2	55	52	46–71	13.81	0.21	9.5	24.1
	2	2	143	141	131–213	2.29	0.09	1.5	23.1
	3	3	8	6	0–32	5.20	0.12	3.2	22.0
	5	3	107	110	100–115	2.89	0.10	1.8	21.8
	6	3	164	191	130–191	4.04	0.16	5.2	23.3
	7	3	234	234	224–234	5.28	0.13	3.1	21.7
						**TOTAL %V_P_**		**24.3**	
Sperm motile *vs.* non-motile*vs.* absent	1	2	54	39	45–84	5.49	0.22	4.1	19.9
	2	2	143	140	132–182	4.46	0.21	3.4	19.4
	3	3	7	21	0–34	2.45	0.14	1.6	19.9
	4	3	79	66	36–93	6.35	0.25	5.9	21.2
	6	3	166	159	134–208	3.48	0.25	5.4	22.1
	7	3	234	234	218–234	2.71	0.14	1.6	18.4
						**TOTAL %V_P_**		**22.0**	
Sperm present (motile andnon-motile) *vs.* absent	2	2	142	140	131–183	3.12	0.11	2.7	5.3
	4	3	54	57	36–100	2.67	0.11	2.8	5.8
						**TOTAL %V_P_**		**5.5**	

1 The QTL # is the order of the QTL peaks from left to right in Figure 1.

2 cM is the location of the highest likelihood score in centimorgans (cM) from the left hand of each chromosome, as determined by CIM; the refined position is by MIM.

3 Range is the span of the QTL, determined by a 95% confidence interval.

4 Max. LOD is the maximum LOD score calculated with CIM (individual QTLs) or MIM (epistatic interactions).

5 The additive effect is half of the difference between the two homozygous classes.

6 %V_P_ is the proportion of the phenotypic variance accounted for by each QTL with the estimated parameters (calculated by taking *R* ^2^*100).

7 %TV_P_ is the proportion of the phenotypic variance accounted for by each QTL given the number of background cofactors (calculated by taking T*R* ^2^*100).

**Table 2:** Epistatic interactions among QTL.

Comparison	Test^1^	Interaction	LOD	Additive Effect^4^	%V_P_ 5
Sperm motile *vs.* non-motile(present and absent)	QTL-QTL	1×6	11.23	0.37	6.6
	QTL-other	1×8^2^	8.69	0.30	17.0
		6×8^2^	3.56	0.20	6.3
		7×8^2^	3.67	0.20	1.6
				**TOTAL %V_P_**	**31.5**
Sperm motile *vs.* non-motile *vs.* absent	QTL-QTL	2×7	3.16	0.32	2.6
	QTL-other	6×9^3^	5.13	0.70	4.6
				**TOTAL %V_P_**	**7.2**

1 The test is either ‘QTL-QTL,’ which identifies epistatic interactions between main-effect QTLs (Figure 1, unboxed numbers; Table 1), or ‘QTL-other,’ which identifies epistatic interactions between QTLs and non-QTL regions of the genome (Figure 1, boxed numbers). Note that there were no significant epistatic effects detected when the phenotype was scored only by sperm presence *vs*. absence.

2 QTL #8 is located at 17 cM on chromosome 3.

3 QTL #9 is located at 197 cM on chromosome 3.

4 The additive effect is half of the difference between the two homozygous classes.

5 %V_P_ is the proportion of the phenotypic variance accounted for by the epistatic interaction.

It should be noted that we observed an interesting phenomenon among the progeny of the crosses: although attached-X F_1_ females are homozygous for a recessive white-eye mutation on their X chromosome, and thus all of these females should have white eyes, approximately 15% (19 out of 125 examined) of interspecies F_1_ females had red eyes, but otherwise appeared normal. This rate is much higher than previously reported for an attached-X *D. melanogaster* stock (approximately 2%, as XXXAA metafemales; [35]). There were no red-eyed females within the attached-X stock, as this phenotype was consistently looked for as it would indicate a breakdown of the attacked-X chromosome; as an added measure, we also did not observe any red-eyed females when intentionally scoring for this phenotype (N = 96). To determine whether these females might be XXX, they were paired with males to assess fertility, as F_1_ hybrid females are typically receptive to mating with males of both species [36] and XXX females are sterile [37], [38]. When crossed separately with either *D. simulans* or *D. mauritiana* males, the red-eyed F_1_ females did not produce any larvae, indicating that they are likely sterile. Therefore, the most plausible explanation for the presence of red-eyed F_1_ females is that they have inherited both the maternal attached-X chromosome and a paternal X chromosome that lacks the recessive white-eye mutation. The single paternally-inherited functional copy of the *white* gene would cause these XXX females to have red eyes, while the presence of three X chromosomes would render these females sterile.

## Discussion

While there have been many recent advances in genetic studies of interspecies hybrid sterility, one major hindrance to the evaluation of the D-M model as a framework for the evolution of hybrid sterility has been the lack of identification of autosomal sterility loci. Previous QTL mapping studies in *Drosophila* have identified few autosomal loci for interspecies hybrid sterility, and all of the identified autosomal loci are of very small effect size, indicating that most of the phenotypic variance was not explained by these QTL. For example, only two autosomal loci for sterility, accounting for less than 5% of the phenotypic variance, were identified for hybrids of *Drosophila santomea* and *D. yakuba* [20]. Chang and Noor [39] identified four autosomal QTL that were capable of acting dominantly on hybrid sterility in *D. persimilis* and *D. pseudoobscura bogotana*, each of small effect. One potential reason for the limited autosomal maps is the very large effect of the X chromosome – recombinant individuals containing particular regions of the X chromosome might display such a consistent sterility phenotype that it hinders the ability to assess the contribution of the rest of the genome.

**Figure 4:**
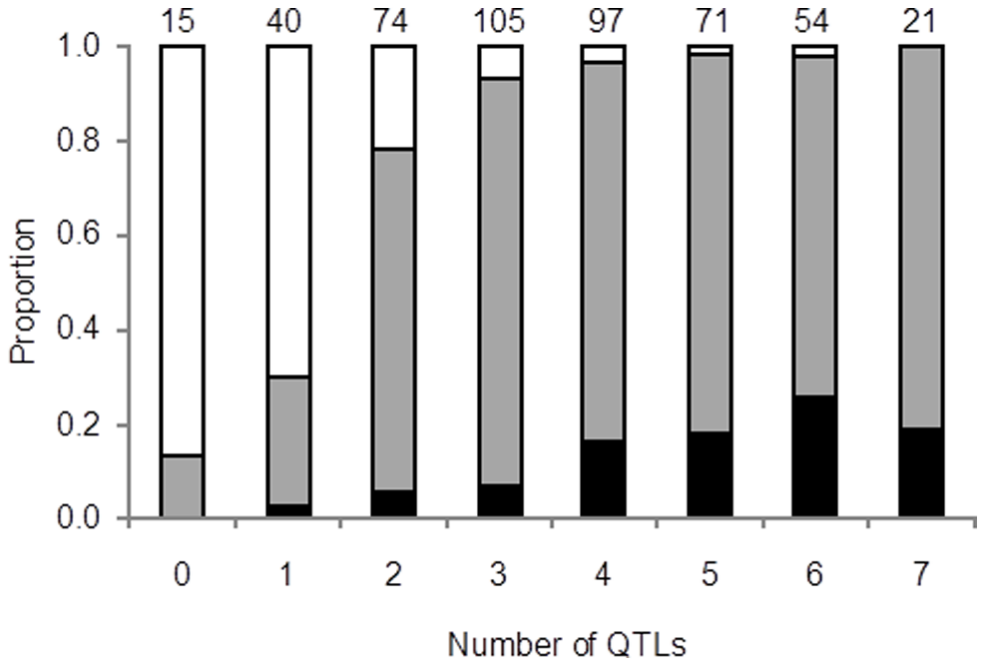
Fertility score per QTL composition. The proportion of individuals with no sperm (black bars), non-motile sperm (grey bars) and motile sperm (white bars), sorted by the number of markers closest to the main-effect QTLs that individual contained, in any combination. The number of total individuals with that number of QTLs is listed above each bar; individuals missing a genotype for any of the seven markers were not included in this figure.

Here, we created hybrid males with a non-recombinant X chromosome and recombinant autosomes, allowing us to more accurately identify the number and location of autosomal loci contributing to hybrid sterility. We identified nine autosomal QTL in total that act dominantly to contribute to interspecies hybrid sterility; as each QTL may contain more than one locus contributing to the phenotype, this is a minimum estimate of the number of genes. These QTL are evenly-distributed across the autosomes, indicating a broad genetic architecture. The more main-effect QTLs an individual contained, the more likely they were to have a sterility phenotype (Figure 4): almost all individuals containing none of the QTLs had motile sperm, while none of the individuals with all seven main-effect QTLs had motile sperm. There was a threshold effect, however, whereby individuals with four to seven of the QTLs had approximately the same odds of a sterility phenotype. While a threshold of sterility is to be expected (once sterile, and individual can’t get ‘more sterile’), the presence of additional QTLs, up to four, increased the likelihood of having non-motile sperm or the absence of sperm, but additional QTLs above four did not increase the incidence of these phenotypes.

A comparison of the main-effect QTLs for the traits we measured gives some indication regarding how the loci within each QTL are impacting fertility. While the power to detect QTL effects can be impacted by the number of individuals in each category, which differed based on how we divided the analysis groups, QTLs #1, 3 and 7 do not appear to contribute to whether sperm is present or absent, as there is no increase in LOD (even if non-significant) for this trait at those QTL locations (blue line, Figure 3). Thus, these QTLs only affect whether the sperm that is produced is motile or non-motile, but do not contribute to the phenotype of sperm presence/absence. In contrast, only the inclusion of the production of sperm (sperm presence or absence; purple and blue lines, Figure 3) yields a significant effect at QTL #4, indicating that this region contributes to sperm production, but not sperm motility. An additional comparison can be made to a previous study that utilized introgressions and found a single genomic region that acted dominantly to induce sterility in *D. simulans-D. mauritiana* hybrids [40]. The significant dominant effect was attributed to a region within recombination map positions 43 and 74 of the second chromosome in *D. simulans*, which overlaps our strongest peak, QTL#1. Thus it is possible that the same region was identified in both Hollocher and Wu [40] and the current study.

Elimination of the variation due to the sex chromosome allowed us to identify QTLs that contribute to up to 56% of the phenotypic variation in autosome-induced sterility. Thus, it appears that hybrid sterility is not caused by a very large number of small-effect autosomal loci, unless these loci are clustered within the QTLs, which is possible as such clustering may be common [41]. Approximately half of the variance we can explain is due to main-effect QTLs (or clusters), which likely cause their effect through interactions with loci on the sex chromosome. The other half is due to epistatic interactions within the autosomes. While it is possible that these interactions may be three-way and also involve a locus on the sex chromosomes, autosome-autosome epistatic interactions were also reported as contributing to sterility in hybrids of *D. persimilis* - *D. pseudoobscura bogotana* [42], *D. buzzatii* - *D. koepferaehouse* [43], and *D. mojavensis* - *D. arizonae* [44], and *Mus musculus musculus* – *M. m. domesticus* [45], [46], making autosome-autosome interactions a distinct possibility in the species pair we present here.

Surprisingly, all of the males from the *D. simulans* backcross lacked motile sperm (Figure 2). These males may be sterile due to an epistatic interaction between the *D. simulans* X chromosome and the *D. mauritiana* Y chromosome, an idea that is supported by studies that have substituted the sex chromosomes between these species, resulting in sterile male offspring [34], [47], [48]. Alternatively, sterility in these males could be caused by interactions between the X chromosome of *D. simulans* and dominant loci on the autosomes of *D. mauritiana*, between the Y chromosome of *D. mauritiana* and dominant loci on the autosomes of *D. simulans*, or between a dominant autosomal locus from *D. mauritiana* and a recessive autosomal locus from *D. simulans*; all three interactions would support the D-M-dominance model. For the X-autosomal and autosomal-autosomal interactions to induce sterility in the *D. simulans* backcross males, there would need to be a very large number of interactions capable of causing sterility, otherwise some of the 266 tested males, by chance, would be expected to not contain the necessary autosomal alleles, and would be fertile. Taking the above QTL mapping results into account, it seems less likely that the observed sterility is due to many autosomal loci, each of very small effect, scattered throughout the genome. Thus, the most likely cause of sterility in these backcross individuals is either an X-Y or Y-autosomal interaction, the latter of which is supported by a study demonstrating that Y substitutions between these species result in sterility [47]. There is not the possibility of an interspecies X-Y interaction in the reciprocal backcross (Figure 1), and the interspecies autosome is recombinant (and thus variable) if we instead consider Y-autosome interactions; either scenario may explain why we obtained some fertile individuals in this cross. It is therefore possible that an X-Y or Y-autosome interaction is the underlying basis for F_1_ hybrid sterility in this species pair.

## Supporting Information

Table S1 **List of microsatellite markers and their primers used for genotyping.**
(DOCX)

Table S2 **Genotype and phenotype data for the backcross (BC)** ***D. mauritiana*** **individuals.**
(DOCX)

Table S3 **Proportion of genotypes for the marker closest to each QTL peak in the backcross** ***D. mauritiana*** **data set, sorted by sperm phenotype.**
(DOCX)
