# Supplementary material for: A Novel Approach Identifying Hybrid Sterility QTL on the Autosomes of Drosophila simulans and D. mauritiana
Source: PLoS One. 2013 Sep 5;8(9):e73325. doi: 10.1371/journal.pone.0073325 (PMC3764152; doi:10.1371/journal.pone.0073325)
Supplement: Table S1 — List of microsatellite markers and their primers used for genotyping. (DOCX) [file pone.0073325.s001.docx]

Table S1. List of microsatellite markers and their primers used for genotyping. Genomic location is relative to the *D. simulans* published genome sequence for the X chromosome and the left and right arms of the second and third chromosomes (2L, 2R, 3L, and 3R, respectively). ^1^ Moehring *et al*. 2004; ^2^ Civetta *et al*. 2002; ^3^ Schug *et al*. 1997; ^4^ McNiven and Moehring 2013.

| **Name** | **Forward primer** | **Reverse primer** | **Genomic Location** |
| --- | --- | --- | --- |
| 2L 770 | GTGCAGCGCCTTTATGTTTT | TGCTCTCGTTGAAAATGTCG | 2L 770995 |
| AC0005889^1^ | GCGTGGCTGGCATATAG | TAAGCCCCCTCGTGTAATTG | 2L 9002412 |
| 2L 11774B | TCCGAGATCCGTGTCTTTCT | CATGTTGCATTTGCCTTGAC | 2L 11775227 |
| Su(h)^2^ | AACGGCTCACCCCTCGATCC | TACTTCTCCATGGCGTCCCG | 2L 14787128 |
| 2L 21651 | TCGCACTTTACGAGGTGTTG | AATGCCAGTTCGGATAGTCG | 2L 21651886 |
| 2R 700 | CTGGAACTGTGGGTGGAAAG | CCCATCTCATCTCCCTTCCT | 2R 700869 |
| Drogpad^3^ | GAAATAGGAATCATTTTGAATGGC | AATTAAAAACAAAAAACCTGAGCG | 2R 4976473 |
| 2R 14938 | CACCCTTACCCTGTTCCTCA | GACTTTCCCCTTTTCCTTGC | 2R 14938944 |
| 2R 15381B | CGGAACCAGCAGAAACTCTAA | TCACAGACCCTCCATTCAAAG | 2R 15381226 |
| 2R 19158 | GCTCACGTTCGTTTATGCTG | CGGTGCAAATTACGACACAG | 2R 19158946 |
| 3L 1457 | TGGAGAGCGGCGTTCCCCTGTGT | TGGGCCACCTGTGGGCGTGGT | 3L 1457712 |
| 3L 3484 | GAGGACAGGCGGTACATGAG | TAGTCCGTGGGCAGTAGCTC | 3L 3484769 |
| 3L 10365 | GACCCGAGAGCATTCTTGAG | GTTTCCCTGCCCAAGAGACAATTA | 3L 10365945 |
| 3L 16008 | CCAAGGGGCAGAAATAGGTA | GGAGCAACAATTGCATCAGA | 3L 16008277 |
| 3R 697 | GGAGATGCCAAACGAAATA | CTCTTTCCGCTCCCCTTA | 3R 697841 |
| 3R 3880 | CCTCCTTGGAATGATCCTCA | ATTATCCAAGTGCGGACGAC | 3R 3880676 |
| 3R 4012 | CGGGTTAATTGGACTTGCAT | CTGGCCAAGTCGAGAAAAAG | 3R 4012692 |
| 3R 17066 | GCGATTGTGTGCGAGTGTAT | GGGGGATTTTGTTTGTCATC | 3R 17066022 |
| 3R 20144 | GAACAAGCCGGCATACAGAT | GTTTAGGCACATTTGGATTGGATT | 3R 20145125 |
| 3R 23001^4^ | TAGCTGCCATCGAGTGTGTC | GTTTTGCGGCTAATGAGAGG | 3R 23002040 |
| X 16836 | GGGCGGAAAGTAGAGAAGGT | GCCCACTGATTTGGCTATGT | X 16836880 |

**References**:

Civetta A, Waldrip-Dail HM, Clark AG (2002) An introgression approach to mapping differences in mating success and sperm competitive ability in *Drosophila simulans* and *D. sechellia*. Genet Res 79: 65-74.

McNiven VTK, Moehring AJ (*in press*). Identification of a genetically linked female preference and male trait. Evolution

Moehring AJ, Li J, Schug MD, Smith SG, DeAngelis M, Mackay TFC, Coyne JA (2004) Quantitative trait loci for sexual isolation between *Drosophila simulans* and *D. mauritiana*. Genetics 167: 1265-1274.

Schug MD, Mackay TFC, Aquadro CF (1997) Low mutation rates of microsatellite loci in *Drosophila melanogaster*. Nat Genet 15: 99-102.
