# Supplementary material for: A Novel Approach Identifying Hybrid Sterility QTL on the Autosomes of Drosophila simulans and D. mauritiana
Source: PLoS One. 2013 Sep 5;8(9):e73325. doi: 10.1371/journal.pone.0073325 (PMC3764152; doi:10.1371/journal.pone.0073325)
Supplement: Table S2 — Genotype and phenotype data for the backcross (BC) D. mauritiana individuals. (DOCX) [file pone.0073325.s002.docx]

**Table S2.** Genotype and phenotype data for the backcross (BC) *D. mauritiana* individuals. ‘Name’ is the name given to each tested individual. ‘Fertility’ is the phenotype scored as 0=no sperm, 1=non-motile sperm, 2=motile sperm. ‘Motility’ is the phenotype scored as 0=non-motile or absent sperm, 1=motile sperm. ‘Sperm’ is the phenotype scored as 0=no sperm, 1=non-motile or motile sperm. Genotypes are listed as 1=heterozygous (*sim*/*mau*), 0=homozygous (*mau*/*mau*), . =missing data.

|  | **Phenotypes** | | | **Chromosome 2 markers** | | | | | | | | | | **Chromosome 3 markers** | | | | | | | | | |
| --- | --- | --- | --- | --- | --- | --- | --- | --- | --- | --- | --- | --- | --- | --- | --- | --- | --- | --- | --- | --- | --- | --- | --- |
| **Name** | **Fertility** | **Motility** | **Sperm** | 770 | AC0005889 | 11774b | *Su(h)* | 21651 | 700 | Drogpad | 14938 | 15381 | 19158 | 1457 | 3484 | 10365 | 16008 | 697 | 3880 | 4012 | 17066 | 21044 | 23001 |
| BCmau2-1 | 1 | 0 | 1 | 0 | 0 | 0 | . | 0 | 0 | 0 | 1 | 1 | 1 | 1 | 0 | 1 | 1 | 0 | 0 | 0 | 0 | 0 | 1 |
| BCmau2-2 | 1 | 0 | 1 | 0 | 0 | 0 | . | 0 | 0 | 0 | 1 | 1 | 1 | 1 | 1 | 1 | 1 | . | 1 | 1 | 1 | 1 | 1 |
| BCmau2-3 | 1 | 0 | 1 | 1 | 1 | 1 | 0 | 1 | 1 | 1 | 1 | 0 | . | 1 | 1 | 1 | 1 | 1 | 1 | 0 | 0 | 0 | 0 |
| BCmau2-2 | 1 | 0 | 1 | . | 1 | 1 | . | 1 | 1 | 1 | 1 | 1 | 1 | 0 | 0 | 1 | . | 1 | . | 0 | 1 | 1 | 1 |
| BCmau2-5 | 1 | 0 | 1 | 0 | 0 | 0 | 0 | 0 | 0 | 1 | 1 | 1 | 1 | 1 | 1 | 1 | 1 | 0 | 0 | . | 1 | 1 | 1 |
| BCmau2-6 | 1 | 0 | 1 | 0 | 0 | 0 | 0 | 1 | 1 | 1 | 0 | 0 | 0 | 1 | 1 | 1 | 0 | 0 | . | 0 | 1 | 1 | 1 |
| BCmau2-7 | 1 | 0 | 1 | . | 0 | 0 | 0 | 0 | 0 | 0 | 1 | 1 | 1 | 0 | 0 | 0 | 0 | 0 | 0 | 0 | 1 | 1 | 1 |
| BCmau2-8 | 1 | 0 | 1 | 0 | 1 | 1 | 0 | 1 | 1 | 1 | 0 | 0 | 0 | 0 | 0 | 0 | 0 | 1 | 0 | 0 | 1 | 1 | 1 |
| BCmau2-9 | 1 | 0 | 1 | 1 | 1 | 1 | 0 | 1 | 1 | 1 | 1 | 1 | 1 | 1 | 1 | 1 | 1 | 1 | 0 | 0 | 1 | 1 | 1 |
| BCmau2-10 | 1 | 0 | 1 | 1 | 1 | 1 | 0 | 1 | 1 | 1 | 0 | 0 | 1 | 0 | 0 | 0 | 0 | 0 | . | 0 | 0 | 0 | 0 |
| BCmau2-11 | 2 | 1 | 1 | 1 | 0 | 0 | . | 0 | 0 | 0 | 0 | 0 | 0 | 0 | 0 | 0 | 0 | 0 | 0 | 0 | 0 | 0 | 0 |
| BCmau2-12 | 1 | 0 | 1 | 1 | 0 | 0 | 0 | 0 | 0 | 0 | 0 | 0 | 0 | 0 | 0 | 0 | 0 | 0 | 0 | 0 | 1 | 1 | 1 |
| BCmau2-13 | 1 | 0 | 1 | 0 | 0 | 1 | 0 | 0 | 0 | 0 | 1 | 1 | 0 | 1 | . | 1 | 0 | 0 | 0 | 0 | 1 | 1 | 1 |
| BCmau2-12 | 1 | 0 | 1 | . | 0 | 1 | 0 | 1 | 1 | 1 | 1 | 1 | 1 | 0 | 0 | 1 | 1 | 1 | 1 | 1 | 1 | 1 | 1 |
| BCmau2-15 | 1 | 0 | 1 | 1 | 1 | 1 | . | 1 | 1 | 1 | 1 | 0 | 1 | 1 | 1 | 1 | . | 1 | 1 | 1 | 1 | 1 | 1 |
| BCmau2-16 | 1 | 0 | 1 | 1 | 1 | 1 | . | 1 | 1 | 1 | 1 | 0 | 1 | 0 | 0 | 0 | 0 | 0 | 0 | 0 | 0 | 0 | 0 |
| BCmau2-17 | 1 | 0 | 1 | . | 1 | 1 | . | 1 | 1 | 1 | 0 | 0 | 0 | 1 | 1 | 0 | 0 | 0 | 0 | 0 | 0 | 0 | 0 |
| BCmau2-18 | 1 | 0 | 1 | 1 | 1 | 1 | 0 | 1 | 1 | 1 | 1 | 1 | 1 | 1 | 1 | 1 | 1 | 0 | 0 | 0 | 1 | 1 | 1 |
| BCmau2-19 | 1 | 0 | 1 | 0 | 0 | 0 | 0 | 0 | 0 | 0 | 0 | 0 | 0 | 1 | 1 | 1 | 0 | 1 | 1 | 1 | 1 | 1 | 1 |
| BCmau2-20 | 0 | 0 | 0 | 0 | 0 | 0 | 0 | 0 | 0 | 1 | 1 | 1 | 1 | 1 | 1 | 1 | 1 | . | 1 | 1 | 1 | 0 | 0 |
| BCmau2-21 | 1 | 0 | 1 | 1 | 1 | 1 | 1 | 1 | 1 | 1 | 1 | 1 | 1 | 1 | 1 | 0 | 0 | 1 | 1 | 1 | 0 | 0 | 0 |
| BCmau2-22 | 1 | 0 | 1 | . | 1 | 1 | 1 | 1 | 1 | 1 | 0 | 0 | . | 1 | 1 | 0 | 0 | . | 1 | 1 | 1 | 1 | 1 |
| BCmau2-23 | 1 | 0 | 1 | 0 | 0 | 0 | 0 | 0 | 0 | 0 | . | 1 | 1 | 0 | 0 | 0 | 0 | 0 | 0 | 0 | 1 | 0 | 0 |
| BCmau2-22 | 2 | 1 | 1 | 1 | 0 | 0 | . | 0 | 0 | 0 | 1 | 1 | 0 | 0 | 0 | . | 0 | . | 0 | 0 | 0 | . | 1 |
| BCmau2-25 | 1 | 0 | 1 | 0 | 0 | 0 | . | 0 | 0 | 0 | 1 | 1 | 1 | 1 | 1 | 0 | 0 | 0 | 1 | 0 | 1 | 0 | 0 |
| BCmau2-26 | 1 | 0 | 1 | 0 | 1 | 1 | . | 1 | 1 | 0 | 0 | 0 | 0 | 0 | 0 | 1 | 0 | 0 | . | 0 | 1 | . | 1 |
| BCmau2-27 | 1 | 0 | 1 | 1 | 1 | 1 | 1 | 0 | 0 | 1 | 0 | 0 | 0 | 1 | . | . | 1 | 1 | 0 | . | 1 | . | 1 |
| BCmau2-28 | 1 | 0 | 1 | 0 | 0 | 0 | 0 | 0 | 0 | 0 | 0 | 0 | . | 1 | . | . | 0 | . | 1 | 0 | 1 | . | 1 |
| BCmau2-29 | 1 | 0 | 1 | 0 | 0 | 0 | . | 0 | 0 | 1 | 0 | 0 | 0 | 1 | 1 | . | 1 | 0 | 1 | 0 | 0 | 0 | 0 |
| BCmau2-30 | 1 | 0 | 1 | 0 | 0 | 0 | 0 | 0 | 0 | 0 | 1 | 1 | 1 | 1 | 1 | . | 0 | 1 | . | . | 0 | 0 | 0 |
| BCmau2-31 | 1 | 0 | 1 | 0 | 0 | 0 | . | 1 | 1 | 1 | . | 1 | 1 | 0 | 0 | 0 | . | 1 | 0 | 0 | 1 | 0 | 1 |
| BCmau2-32 | 2 | 1 | 1 | 0 | 0 | 0 | . | 0 | 0 | 0 | 0 | 0 | 0 | 0 | . | . | . | 1 | . | . | 0 | 1 | 0 |
| BCmau2-33 | 1 | 0 | 1 | 0 | 0 | 0 | 0 | 0 | 0 | 0 | 0 | 0 | 1 | 0 | 0 | . | 1 | 1 | . | 1 | 0 | 0 | 0 |
| BCmau2-32 | 1 | 0 | 1 | 0 | 1 | 1 | . | 1 | 1 | 1 | 0 | 0 | 0 | 0 | 0 | 0 | 0 | . | 0 | 0 | 0 | 0 | 1 |
| BCmau2-35 | 1 | 0 | 1 | . | 1 | 1 | 0 | 1 | 1 | 1 | 0 | 0 | . | 1 | 0 | 0 | 1 | 1 | 0 | 1 | 0 | 1 | 0 |
| BCmau2-36 | 1 | 0 | 1 | 0 | 0 | 0 | 0 | 1 | 1 | 1 | 0 | 0 | 0 | 1 | 1 | 1 | 1 | . | 1 | 1 | 1 | 0 | 0 |
| BCmau2-37 | 1 | 0 | 1 | . | 0 | 0 | 0 | 0 | 0 | 0 | 1 | 1 | 1 | 1 | . | 0 | 0 | 0 | . | 1 | 1 | 1 | 1 |
| BCmau2-38 | 1 | 0 | 1 | 1 | 1 | 1 | . | 1 | 1 | 1 | 0 | 0 | 0 | 0 | 0 | 0 | 0 | 1 | . | 1 | 0 | 0 | 0 |
| BCmau2-39 | 1 | 0 | 1 | 0 | 0 | 0 | 0 | 1 | 1 | 1 | 0 | 0 | 0 | 1 | 1 | . | 0 | 1 | . | 1 | 1 | 1 | 0 |
| BCmau2-20 | 0 | 0 | 0 | . | 0 | 1 | . | 1 | 1 | 1 | . | 1 | 1 | 1 | 1 | 1 | 0 | 0 | 0 | 0 | 1 | 1 | 1 |
| BCmau2-21 | 0 | 0 | 0 | 1 | 0 | 0 | 0 | 1 | 1 | 1 | 0 | 0 | 0 | 0 | 0 | . | . | 1 | 1 | 1 | 0 | 0 | 0 |
| BCmau2-22 | 1 | 0 | 1 | 1 | 0 | 0 | . | 0 | 0 | 0 | 0 | 0 | 0 | 0 | 0 | 0 | 0 | 0 | 0 | 0 | 1 | 1 | 1 |
| BCmau2-23 | 1 | 0 | 1 | 0 | 0 | 1 | . | 1 | 1 | 1 | 0 | 0 | 0 | 0 | 0 | . | 0 | 1 | 1 | 1 | 0 | 1 | 1 |
| BCmau2-22 | 0 | 0 | 0 | 1 | 1 | 1 | 0 | 1 | 1 | 1 | 1 | 1 | 1 | 1 | 0 | . | 1 | 1 | 1 | 1 | 1 | 1 | 1 |
| BCmau2-25 | 0 | 0 | 0 | 0 | 0 | 0 | . | 1 | 1 | 1 | 0 | 1 | 1 | 1 | . | . | 0 | 1 | . | 0 | . | . | 0 |
| BCmau2-26 | 1 | 0 | 1 | 1 | 1 | 0 | 0 | 1 | 1 | 1 | 0 | 0 | 0 | 0 | 1 | 1 | 1 | 1 | 1 | . | 1 | 1 | 1 |
| BCmau2-27 | 0 | 0 | 0 | 0 | 0 | . | . | . | 1 | 0 | 0 | 1 | . | 0 | 1 | . | . | . | . | . | 1 | . | 1 |
| BCmau2-28 | 0 | 0 | 0 | 0 | 1 | 0 | 0 | 1 | 1 | 0 | 0 | 0 | 0 | 1 | 1 | 1 | 1 | 1 | 0 | 0 | 1 | 1 | 1 |
| BCmau2-29 | 1 | 0 | 1 | 1 | 1 | 1 | 1 | 1 | 1 | 1 | 1 | 1 | 1 | 0 | 0 | 0 | 0 | 0 | 0 | 0 | 0 | 0 | 0 |
| BCmau2-50 | 1 | 0 | 1 | . | 0 | 0 | 0 | 0 | 0 | 0 | 1 | . | 1 | 1 | . | . | . | 1 | . | 1 | 0 | 0 | 0 |
| BCmau2-51 | 0 | 0 | 0 | . | 0 | 0 | 0 | 0 | 0 | 0 | 1 | 1 | 1 | 1 | . | . | 0 | . | 1 | 1 | 1 | 1 | 1 |
| BCmau2-52 | 2 | 1 | 1 | 1 | 0 | 0 | 0 | 1 | 1 | 1 | 0 | 0 | 0 | 0 | . | 0 | 0 | 0 | 0 | 1 | 0 | 0 | 0 |
| BCmau2-53 | 1 | 0 | 1 | 1 | 1 | 1 | . | 1 | 1 | 1 | 1 | 1 | . | 0 | 0 | . | 1 | 0 | 0 | . | 0 | 0 | 0 |
| BCmau2-52 | 1 | 0 | 1 | 1 | 1 | 1 | 1 | 0 | 0 | 1 | 0 | 0 | . | 1 | 1 | 1 | 0 | 1 | 1 | 1 | 0 | 0 | 0 |
| BCmau2-55 | 1 | 0 | 1 | 1 | 1 | 0 | 0 | 0 | 0 | 0 | 0 | 1 | . | 1 | 1 | 1 | 0 | 0 | 0 | 0 | 1 | 0 | 0 |
| BCmau2-56 | 1 | 0 | 1 | 1 | 1 | 1 | 1 | 1 | 1 | 1 | 0 | 0 | . | 1 | 1 | 0 | 0 | 1 | 1 | 1 | 1 | 0 | 0 |
| BCmau2-57 | 0 | 0 | 0 | . | 1 | 1 | 1 | 1 | 1 | 1 | 0 | 0 | 0 | 1 | 1 | . | 0 | 1 | . | 0 | 1 | 0 | . |
| BCmau2-58 | 1 | 0 | 1 | 0 | 0 | 0 | 0 | 0 | 0 | 0 | 1 | 1 | . | . | . | . | 1 | 1 | 1 | 1 | 0 | . | . |
| BCmau2-59 | 2 | 1 | 1 | 0 | 0 | 0 | 0 | 0 | 0 | 0 | 0 | 1 | . | 0 | 0 | . | 1 | 0 | 0 | 0 | 0 | 0 | 0 |
| BCmau2-60 | 0 | 0 | 0 | 1 | 1 | 1 | . | 0 | 0 | 0 | 0 | 0 | 0 | 1 | 1 | . | 0 | 1 | 1 | 1 | 1 | 1 | 1 |
| BCmau2-61 | 1 | 0 | 1 | 0 | 0 | 1 | 0 | 0 | 0 | 0 | 0 | 0 | 0 | 1 | 1 | . | 1 | 1 | 1 | . | 1 | 1 | 0 |
| BCmau2-62 | 1 | 0 | 1 | 0 | 0 | 0 | 0 | 0 | 0 | 0 | 0 | 1 | 1 | 1 | . | 0 | 0 | 1 | 1 | . | 0 | 0 | 0 |
| BCmau2-63 | 1 | 0 | 1 | 1 | 1 | 0 | 0 | 1 | 1 | 1 | 0 | 0 | 1 | 0 | 0 | . | 1 | 1 | 1 | 0 | 0 | 0 | 1 |
| BCmau2-62 | 1 | 0 | 1 | 0 | 0 | 0 | 0 | 1 | 1 | 1 | 0 | 0 | 0 | 0 | 0 | 0 | 1 | . | 1 | . | 1 | 1 | 1 |
| BCmau2-65 | 1 | 0 | 1 | 0 | 0 | 0 | 0 | . | 0 | 0 | 0 | 0 | 0 | 0 | 0 | 0 | 1 | 1 | 0 | 0 | 1 | 1 | 1 |
| BCmau2-66 | 1 | 0 | 1 | 0 | 1 | 1 | 0 | 1 | 1 | 1 | 1 | 1 | 1 | 1 | 1 | 1 | 0 | 0 | . | 0 | 1 | 1 | 1 |
| BCmau2-67 | 0 | 0 | 0 | . | 1 | 1 | . | 0 | 0 | 1 | 1 | 0 | 1 | 1 | 1 | 1 | 1 | . | 0 | 0 | 1 | 1 | 1 |
| BCmau2-68 | 1 | 0 | 1 | 1 | 0 | 0 | 0 | 0 | 0 | 0 | 1 | 1 | . | 0 | 0 | 1 | 0 | 1 | 0 | 0 | 0 | 0 | 0 |
| BCmau2-69 | 1 | 0 | 1 | . | 1 | 1 | . | 1 | 1 | 1 | 1 | 0 | 0 | 1 | 1 | 1 | 1 | 1 | 1 | 0 | 1 | 0 | 0 |
| BCmau2-70 | 2 | 1 | 1 | 0 | 0 | 0 | 0 | 0 | 0 | 0 | 0 | 0 | . | 0 | 0 | 0 | 0 | 0 | 0 | 0 | 0 | 1 | 0 |
| BCmau2-71 | 1 | 0 | 1 | 1 | 1 | 1 | . | 0 | 0 | 0 | 0 | 0 | 0 | 1 | 1 | 1 | 0 | 0 | 0 | 1 | 0 | 1 | 1 |
| BCmau2-72 | 0 | 0 | 0 | 0 | 0 | . | 1 | 1 | 1 | 1 | 0 | 1 | 1 | . | 0 | . | . | . | 1 | . | 1 | . | 0 |
| BCmau2-73 | 1 | 0 | 1 | 1 | 1 | 1 | . | 1 | 1 | 1 | 1 | 1 | . | 0 | 0 | 1 | 1 | 1 | 1 | 0 | 0 | 0 | 0 |
| BCmau2-72 | 0 | 0 | 0 | 1 | 1 | 1 | 1 | 1 | 1 | 1 | 1 | 1 | 1 | 1 | 1 | 1 | 1 | 0 | 0 | 0 | 0 | 0 | 0 |
| BCmau2-75 | 1 | 0 | 1 | 1 | 1 | 1 | 0 | 1 | 1 | 1 | 0 | 0 | 0 | 0 | 0 | 0 | 0 | 0 | 0 | 0 | 0 | 0 | 0 |
| BCmau2-76 | 1 | 0 | 1 | 0 | 1 | 1 | 1 | 0 | 0 | 0 | 0 | 0 | 0 | 1 | 1 | 1 | 0 | 0 | 0 | 0 | 1 | 1 | 1 |
| BCmau2-77 | 1 | 0 | 1 | 0 | 1 | 1 | . | 1 | 1 | 1 | 1 | 1 | 1 | 1 | 1 | 0 | 0 | 0 | 0 | 0 | 0 | 0 | 1 |
| BCmau2-78 | 1 | 0 | 1 | 0 | 0 | 0 | . | 0 | 0 | 0 | 0 | 0 | 0 | 1 | . | 1 | 1 | 1 | 1 | 1 | 1 | 1 | 1 |
| BCmau2-79 | 1 | 0 | 1 | . | 1 | 1 | . | 1 | 1 | 1 | 0 | 0 | 0 | 1 | 1 | 0 | 0 | 1 | 1 | 1 | 0 | 0 | 0 |
| BCmau2-80 | 1 | 0 | 1 | 0 | 0 | 0 | . | 0 | 0 | 1 | 1 | 1 | 1 | 1 | 1 | 0 | 0 | . | 1 | 0 | 1 | 1 | 1 |
| BCmau2-81 | 1 | 0 | 1 | . | 0 | 0 | 0 | 0 | 0 | 0 | 1 | 1 | 1 | 0 | 0 | 0 | . | 0 | 0 | 0 | 0 | 0 | 0 |
| BCmau2-82 | 0 | 0 | 0 | 1 | 1 | . | . | . | 0 | 0 | 0 | 0 | 0 | 0 | 0 | 0 | 0 | 1 | . | 0 | 1 | 1 | 1 |
| BCmau2-83 | 0 | 0 | 0 | 1 | 1 | 1 | 1 | 1 | . | 1 | 1 | 1 | 1 | 1 | 1 | 1 | 1 | 1 | 1 | 1 | 1 | 1 | 1 |
| BCmau2-82 | 1 | 0 | 1 | . | 1 | 1 | . | . | 0 | 0 | 1 | 1 | 1 | 0 | 0 | 0 | 0 | 0 | 0 | 0 | 1 | 1 | 1 |
| BCmau2-85 | 1 | 0 | 1 | 0 | 1 | 1 | 1 | 1 | 1 | 1 | 0 | 0 | 0 | 0 | 0 | 0 | 1 | 1 | . | 0 | 0 | 0 | 0 |
| BCmau2-86 | 1 | 0 | 1 | 0 | 1 | 1 | . | 1 | 1 | 1 | 1 | 1 | 0 | 0 | 0 | 0 | 0 | 0 | 0 | 0 | 0 | 0 | 1 |
| BCmau2-87 | 1 | 0 | 1 | . | 1 | 1 | . | 1 | 1 | 1 | 0 | 0 | 0 | 1 | 1 | 0 | 0 | 1 | 1 | 1 | 1 | 1 | 1 |
| BCmau2-88 | 1 | 0 | 1 | 0 | 0 | 0 | 0 | 0 | 0 | 0 | 1 | 1 | 0 | 0 | 0 | 0 | 0 | . | 1 | 1 | 1 | 1 | 1 |
| BCmau2-89 | 1 | 0 | 1 | 1 | 1 | 0 | 0 | 0 | 0 | 1 | 1 | 1 | 1 | 0 | 0 | 0 | 0 | 0 | 0 | 0 | 1 | 1 | 1 |
| BCmau2-90 | 0 | 0 | 0 | 0 | 1 | 1 | 1 | 1 | 1 | 1 | 1 | 1 | 1 | 0 | 0 | 0 | 1 | 1 | 1 | 1 | 1 | 0 | 0 |
| BCmau2-91 | 1 | 0 | 1 | 1 | 1 | 1 | . | 1 | 1 | 1 | 0 | 0 | . | 0 | 0 | . | 1 | 1 | . | 1 | 1 | 1 | 1 |
| BCmau2-92 | 2 | 1 | 1 | 1 | 0 | 0 | 0 | 0 | 0 | 0 | 0 | 1 | . | 0 | 0 | 0 | 0 | . | 1 | 0 | 0 | 0 | 0 |
| BCmau2-93 | 1 | 0 | 1 | 1 | 0 | 0 | . | 0 | 0 | 0 | 0 | 1 | 1 | 1 | . | 1 | 1 | 1 | 1 | 0 | 1 | 0 | 0 |
| BCmau2-92 | 2 | 1 | 1 | 1 | 1 | 0 | 0 | 0 | 0 | 0 | 1 | 1 | 1 | 0 | 0 | 0 | 0 | 0 | . | 1 | 0 | 0 | 0 |
| BCmau2-95 | 1 | 0 | 1 | 0 | 1 | 1 | . | 1 | . | 1 | 1 | 1 | 1 | 0 | 0 | 0 | . | 0 | 0 | 0 | 1 | 1 | 1 |
| BCmau2-96 | 1 | 0 | 1 | 1 | 0 | 0 | 0 | 0 | 0 | . | . | 1 | 1 | 0 | . | . | 0 | . | . | . | 1 | 1 | 1 |
| BCmau3-1 | 2 | 1 | 1 | 0 | 1 | 0 | 0 | . | 0 | 0 | 1 | 0 | 0 | 0 | 0 | 0 | 1 | 1 | 1 | 1 | 1 | 0 | 0 |
| BCmau3-2 | 1 | 0 | 1 | 1 | . | 1 | . | 1 | 1 | 1 | 1 | . | 1 | 0 | 0 | 1 | 0 | 0 | 0 | 0 | 0 | 0 | 0 |
| BCmau3-3 | 1 | 0 | 1 | 1 | 1 | 0 | 0 | 0 | 0 | 0 | 0 | 0 | 0 | 0 | 0 | 1 | 1 | 1 | 1 | 1 | 1 | 1 | 1 |
| BCmau3-2 | 2 | 1 | 1 | 0 | 0 | 0 | 0 | 0 | 0 | 0 | 1 | . | 0 | 1 | 1 | 1 | 0 | 0 | 0 | 0 | 0 | 0 | 0 |
| BCmau3-5 | 1 | 0 | 1 | 0 | 0 | 0 | 0 | 0 | 0 | 0 | 1 | 1 | 1 | 1 | 1 | 0 | 0 | 0 | 0 | 0 | 0 | 1 | 1 |
| BCmau3-6 | 0 | 0 | 0 | 0 | 1 | 1 | 1 | 1 | 1 | 1 | 0 | 0 | 0 | 1 | 1 | 1 | 1 | 1 | 0 | 0 | 1 | 0 | 0 |
| BCmau3-7 | 2 | 1 | 1 | 0 | 0 | 0 | 0 | 0 | 0 | 0 | 1 | 0 | 0 | 1 | 1 | 1 | 1 | 0 | 0 | 0 | 0 | 0 | 0 |
| BCmau3-8 | 2 | 1 | 1 | 1 | 1 | 0 | 0 | 0 | 0 | 0 | 0 | 0 | 1 | 1 | 1 | 0 | 0 | 0 | 0 | 0 | 0 | 1 | 1 |
| BCmau3-9 | 1 | 0 | 1 | 0 | 0 | 0 | 0 | . | 0 | 0 | 0 | 1 | 1 | 0 | 0 | 0 | 1 | 1 | 1 | 1 | 1 | 0 | 1 |
| BCmau3-10 | 0 | 0 | 0 | 1 | 1 | 1 | 1 | 1 | 1 | 1 | 0 | 1 | 1 | 1 | 1 | 0 | 0 | . | 0 | 0 | 0 | 1 | 1 |
| BCmau3-11 | 0 | 0 | 0 | 0 | 0 | 0 | 0 | . | 0 | 1 | 0 | 1 | 0 | 1 | 1 | 1 | 1 | 1 | 1 | 1 | 1 | 1 | 1 |
| BCmau3-12 | 1 | 0 | 1 | 1 | 1 | 1 | 1 | 1 | 1 | 1 | 0 | . | 0 | 1 | 0 | 0 | 0 | 0 | 0 | 0 | 1 | 1 | 1 |
| BCmau3-13 | 1 | 0 | 1 | 1 | 1 | 1 | 0 | . | 1 | 0 | 0 | 0 | 0 | 1 | 1 | 1 | 1 | 1 | 1 | 1 | 0 | 1 | 1 |
| BCmau3-12 | 0 | 0 | 0 | 1 | 1 | 1 | 0 | 1 | 1 | 1 | 0 | 1 | 1 | 1 | 1 | 1 | 1 | 1 | 1 | 1 | 1 | 1 | 1 |
| BCmau3-15 | 1 | 0 | 1 | 0 | . | 1 | 1 | . | 1 | 1 | 1 | 0 | 0 | 0 | 0 | 0 | 1 | 1 | 1 | 1 | 1 | 0 | 0 |
| BCmau3-16 | 1 | 0 | 1 | 1 | . | 1 | 0 | . | 0 | 0 | 0 | 1 | 1 | 0 | 0 | 0 | 1 | 1 | 1 | 1 | 1 | 1 | 1 |
| BCmau3-17 | 0 | 0 | 0 | 1 | 1 | 1 | 1 | 1 | 1 | 0 | 0 | 0 | 0 | 1 | 1 | 1 | 1 | . | 0 | 0 | 0 | 0 | 0 |
| BCmau3-18 | 1 | 0 | 1 | 1 | 1 | 1 | 1 | 1 | . | 1 | 0 | 0 | 0 | 1 | . | 1 | . | 1 | 1 | 1 | 1 | 1 | 1 |
| BCmau3-19 | 0 | 0 | 0 | 1 | 0 | 0 | 0 | 0 | 0 | 1 | . | 1 | 1 | 1 | 1 | 1 | 0 | 1 | 1 | 1 | 0 | 0 | 0 |
| BCmau3-20 | 1 | 0 | 1 | 0 | 0 | 0 | 0 | 0 | 0 | 0 | 0 | 0 | 0 | 0 | 0 | . | . | 1 | 1 | 1 | 0 | 0 | 0 |
| BCmau3-21 | 0 | 0 | 0 | 0 | 0 | 0 | . | 0 | 0 | . | 0 | . | 0 | 1 | . | . | . | 0 | . | . | 1 | 1 | 1 |
| BCmau3-22 | 0 | 0 | 0 | 1 | 1 | 1 | 1 | 1 | 1 | 1 | 0 | 0 | 0 | 1 | 1 | 0 | 0 | 1 | 1 | 1 | 1 | 1 | 1 |
| BCmau3-23 | 1 | 0 | 1 | 1 | 1 | 1 | 0 | 0 | 0 | 0 | 0 | 0 | 1 | . | . | . | . | 1 | . | . | 0 | . | . |
| BCmau3-22 | 1 | 0 | 1 | 1 | 1 | 1 | 1 | 1 | 1 | 1 | 1 | . | 0 | 1 | 1 | 1 | 1 | 1 | 0 | 0 | 0 | 1 | 0 |
| BCmau3-25 | 0 | 0 | 0 | 0 | 1 | 1 | 1 | 1 | 1 | 1 | 0 | 1 | 1 | 1 | 1 | 1 | 1 | 1 | 1 | 1 | 1 | 0 | 0 |
| BCmau3-26 | 1 | 0 | 1 | 0 | 0 | 0 | 0 | 1 | 1 | 1 | 1 | 1 | 0 | 0 | 0 | 0 | 0 | 1 | 1 | 1 | 1 | 0 | 0 |
| BCmau3-27 | 1 | 0 | 1 | 1 | 0 | . | 1 | . | 1 | . | 0 | 1 | 1 | 0 | 0 | 0 | 0 | 1 | 1 | 0 | 0 | 1 | 1 |
| BCmau3-28 | 1 | 0 | 1 | 1 | 1 | 1 | 1 | 0 | 1 | 0 | 0 | 0 | 0 | 0 | 0 | 0 | 0 | 0 | 1 | 1 | 0 | 0 | 1 |
| BCmau3-29 | 0 | 0 | 0 | 1 | 1 | 1 | 1 | . | 0 | 0 | 0 | . | 1 | 1 | 1 | 1 | 1 | 0 | 0 | 0 | 0 | 1 | 1 |
| BCmau3-30 | 2 | 1 | 1 | 1 | 0 | 0 | 0 | . | 0 | 0 | 0 | . | 0 | 0 | 0 | 1 | 1 | . | 1 | 1 | 1 | 1 | 0 |
| BCmau3-31 | 1 | 0 | 1 | 1 | 0 | 0 | 0 | 0 | 1 | 1 | 0 | . | 1 | 1 | 1 | 1 | 1 | 0 | 0 | 0 | 0 | 0 | 0 |
| BCmau3-32 | 1 | 0 | 1 | 1 | . | 1 | 0 | . | 1 | 1 | 0 | 0 | 0 | 1 | 1 | 1 | 0 | 0 | 0 | 0 | 0 | 1 | 1 |
| BCmau3-33 | 1 | 0 | 1 | 0 | 1 | 1 | 1 | 1 | 1 | 1 | . | . | 0 | 0 | 0 | 0 | 1 | 0 | 0 | 0 | 0 | 0 | 0 |
| BCmau3-32 | 0 | 0 | 0 | 1 | 1 | . | 1 | 0 | 0 | 0 | . | . | 0 | 0 | 0 | 1 | 1 | 1 | 1 | 1 | 0 | 1 | 1 |
| BCmau3-35 | 1 | 0 | 1 | 1 | 1 | . | 0 | 1 | 1 | 1 | . | 1 | 1 | 0 | . | 0 | . | 1 | . | . | 0 | . | . |
| BCmau3-36 | 2 | 1 | 1 | 1 | 1 | 1 | 0 | 0 | 0 | 0 | 0 | 0 | 0 | 0 | 0 | 1 | 0 | 0 | 0 | 0 | 0 | 0 | 0 |
| BCmau3-37 | 1 | 0 | 1 | 0 | 0 | 0 | 1 | 0 | 0 | 1 | 1 | 1 | 1 | 0 | 0 | 0 | 1 | 1 | 1 | . | 1 | 0 | 0 |
| BCmau3-38 | 1 | 0 | 1 | 0 | 1 | 1 | 1 | 1 | 1 | 1 | 0 | . | 1 | 0 | 0 | 1 | 0 | 0 | 0 | 0 | 0 | 0 | 0 |
| BCmau3-39 | 1 | 0 | 1 | 1 | 1 | 1 | 1 | . | 1 | 1 | 0 | 0 | 0 | 0 | 0 | 1 | 0 | 0 | 0 | 0 | 0 | 0 | 0 |
| BCmau3-20 | 1 | 0 | 1 | 0 | 0 | 0 | 0 | 0 | 0 | 0 | 0 | . | 1 | . | . | 0 | . | 1 | . | . | 0 | . | . |
| BCmau3-21 | 1 | 0 | 1 | 1 | 1 | 1 | 1 | 1 | 1 | 1 | 0 | . | 1 | 0 | 0 | 0 | 1 | 1 | 0 | 0 | 0 | 1 | 1 |
| BCmau3-22 | 2 | 1 | 1 | 1 | 0 | 0 | 0 | 0 | 0 | 0 | 0 | 0 | 0 | 0 | 0 | 1 | 0 | 0 | 1 | 0 | 0 | 0 | 0 |
| BCmau3-23 | 0 | 0 | 0 | 1 | 0 | 0 | 0 | 1 | 1 | 1 | 0 | . | 0 | 0 | 0 | 1 | 1 | 0 | 0 | 0 | 0 | 1 | 1 |
| BCmau3-22 | 0 | 0 | 0 | 0 | 0 | 0 | 0 | 0 | 1 | 0 | 1 | . | 1 | 0 | 1 | 1 | 0 | 0 | 0 | 0 | 0 | 0 | 0 |
| BCmau3-25 | 1 | 0 | 1 | 0 | 0 | 1 | 1 | . | 1 | 1 | 0 | . | 0 | 0 | 1 | 1 | 0 | 1 | 1 | 1 | 1 | 1 | 1 |
| BCmau3-26 | 1 | 0 | 1 | 1 | 0 | 0 | 0 | 0 | 0 | 0 | 1 | . | 1 | 1 | 1 | 1 | 1 | 0 | 0 | 0 | 0 | 0 | 0 |
| BCmau3-27 | 1 | 0 | 1 | 0 | 0 | 0 | 0 | 0 | 0 | 0 | 0 | 1 | 1 | 0 | 0 | 0 | 0 | 0 | 1 | . | 1 | 1 | 0 |
| BCmau3-28 | 1 | 0 | 1 | 0 | . | 1 | 1 | . | 1 | 1 | 0 | 0 | 0 | 0 | 0 | 0 | 0 | 0 | 0 | . | 0 | 1 | 1 |
| BCmau3-29 | 1 | 0 | 1 | 0 | 1 | 1 | 1 | 1 | 1 | 0 | 1 | 1 | 1 | 0 | 0 | 0 | 0 | 0 | 0 | 0 | 0 | 1 | 0 |
| BCmau3-50 | 1 | 0 | 1 | 1 | 1 | 1 | 1 | 1 | 1 | . | 0 | 1 | 1 | 1 | 1 | 0 | 0 | 0 | 0 | 0 | 0 | 1 | 1 |
| BCmau3-51 | 1 | 0 | 1 | 0 | 0 | 0 | 1 | 1 | 1 | 1 | 0 | 0 | 0 | 0 | 0 | 0 | 0 | 1 | 1 | 1 | 1 | 1 | 1 |
| BCmau3-52 | 1 | 0 | 1 | 1 | 0 | 0 | 0 | 0 | 0 | 0 | 1 | 1 | 0 | 1 | 0 | 0 | 0 | 0 | 1 | 0 | 0 | 1 | 1 |
| BCmau3-53 | 1 | 0 | 1 | 1 | 0 | 0 | 0 | 0 | 0 | 0 | 0 | 1 | 1 | 1 | 1 | 1 | 1 | 1 | 1 | 1 | 1 | 1 | 0 |
| BCmau3-52 | 1 | 0 | 1 | 1 | 1 | 1 | 0 | 0 | 0 | 0 | 0 | 0 | 0 | 0 | 0 | 0 | 0 | 1 | 1 | 1 | 1 | 1 | 1 |
| BCmau3-55 | 1 | 0 | 1 | 1 | 1 | 1 | 1 | . | 1 | 1 | 1 | 1 | 1 | 0 | 0 | 0 | 0 | 0 | 0 | 0 | 0 | 0 | 1 |
| BCmau3-56 | 0 | 0 | 0 | 1 | 1 | . | 0 | . | 0 | 0 | 0 | 0 | 0 | . | . | . | . | 1 | . | . | 1 | . | . |
| BCmau3-57 | 1 | 0 | 1 | 0 | 0 | 0 | 0 | 0 | 0 | 0 | . | . | 1 | 0 | 0 | 1 | . | . | 1 | . | 1 | 1 | 1 |
| BCmau3-58 | 0 | 0 | 0 | 0 | 0 | 1 | 1 | . | 1 | 1 | 1 | 1 | 0 | 1 | 0 | 0 | 0 | 1 | 1 | 1 | 0 | 0 | 0 |
| BCmau3-59 | 1 | 0 | 1 | 0 | 0 | 0 | 0 | . | 0 | . | 0 | 0 | 0 | 0 | 0 | 1 | 1 | 1 | 1 | 1 | 1 | 1 | 1 |
| BCmau3-60 | 1 | 0 | 1 | 1 | 0 | 0 | 0 | 0 | 1 | . | 1 | . | 1 | 1 | 1 | 1 | 1 | 1 | 1 | 1 | 1 | 1 | 1 |
| BCmau3-61 | 1 | 0 | 1 | 0 | 0 | 0 | 0 | . | 0 | 0 | 0 | 0 | 0 | 1 | 1 | 0 | 1 | 1 | 1 | 1 | 0 | 1 | 1 |
| BCmau3-62 | 1 | 0 | 1 | 1 | 1 | . | 1 | 0 | 1 | 1 | 1 | 0 | 0 | 0 | 0 | 0 | 1 | 1 | 1 | . | 1 | 1 | 0 |
| BCmau3-63 | 0 | 0 | 0 | 1 | 1 | 0 | 1 | . | 1 | 1 | . | . | 1 | 1 | 1 | 1 | 1 | . | 1 | . | 1 | 0 | 0 |
| BCmau3-62 | 1 | 0 | 1 | 1 | 0 | 0 | 0 | 0 | 0 | 0 | 1 | . | 1 | 1 | 1 | 1 | 0 | 1 | 1 | 1 | 1 | 1 | 1 |
| BCmau3-65 | 1 | 0 | 1 | 0 | 0 | 1 | 1 | 1 | 1 | . | 0 | 0 | 0 | 0 | 0 | 1 | 1 | 1 | 1 | 1 | 1 | 0 | 0 |
| BCmau3-66 | 1 | 0 | 1 | 0 | 1 | 1 | 0 | 0 | 0 | . | 1 | 1 | 1 | 1 | 1 | 1 | 0 | 0 | 0 | 0 | 0 | 0 | 0 |
| BCmau3-67 | 1 | 0 | 1 | 0 | 0 | . | 1 | . | 1 | 1 | 0 | 0 | 0 | 1 | 1 | 1 | 1 | 1 | 1 | 1 | 0 | 0 | 0 |
| BCmau3-68 | 0 | 0 | 0 | 1 | 1 | 1 | 1 | 0 | 1 | 1 | 1 | 1 | 1 | 0 | 0 | 1 | 1 | 1 | 1 | 1 | 1 | 0 | 0 |
| BCmau3-69 | 1 | 0 | 1 | 1 | 1 | 1 | 1 | 1 | 0 | 0 | 0 | 1 | 0 | 1 | 1 | 1 | 0 | 0 | 0 | 0 | 0 | 1 | 1 |
| BCmau3-70 | 0 | 0 | 0 | . | 0 | 0 | 0 | 1 | 0 | 1 | 1 | 1 | 1 | 1 | 1 | 1 | 0 | 1 | 1 | 1 | 1 | 1 | 1 |
| BCmau3-71 | 1 | 0 | 1 | 0 | 0 | 0 | 1 | 1 | 0 | 1 | 1 | 1 | 1 | 1 | 1 | 0 | 0 | 0 | 0 | 0 | 0 | 0 | 0 |
| BCmau3-72 | 0 | 0 | 0 | 0 | 0 | 0 | 0 | 0 | . | 1 | 0 | 1 | 1 | 1 | 1 | 1 | 1 | . | 0 | 0 | 0 | 1 | 1 |
| BCmau3-73 | 1 | 0 | 1 | 0 | 1 | 1 | 1 | 1 | . | 1 | 1 | 0 | 1 | 1 | 1 | 1 | 0 | 0 | 0 | 0 | 0 | 1 | 1 |
| BCmau3-72 | 1 | 0 | 1 | 0 | 0 | 0 | 0 | 1 | . | 1 | 1 | 1 | 1 | 0 | 1 | 1 | 1 | . | 1 | 1 | 1 | 1 | 1 |
| BCmau3-75 | 1 | 0 | 1 | 1 | 1 | 1 | 1 | . | . | 1 | 0 | . | 0 | 1 | 1 | 0 | 0 | 0 | 0 | 0 | 0 | 1 | 1 |
| BCmau3-76 | 1 | 0 | 1 | 0 | 0 | 1 | 1 | . | . | 1 | 0 | . | 0 | 1 | 1 | 1 | 1 | 1 | 1 | 1 | 1 | 1 | 1 |
| BCmau3-77 | 2 | 1 | 1 | 0 | . | 0 | 1 | 0 | . | 0 | 0 | 0 | 0 | 0 | 0 | 0 | 0 | 1 | 0 | 0 | 0 | 0 | 0 |
| BCmau3-78 | 1 | 0 | 1 | 0 | 1 | 1 | 1 | . | . | 1 | 0 | . | 0 | 0 | 0 | 0 | 0 | 0 | 0 | 1 | 0 | 1 | 1 |
| BCmau3-79 | 0 | 0 | 0 | 1 | 1 | 1 | 1 | 1 | . | 1 | 1 | 1 | 0 | 1 | 1 | 1 | 1 | 1 | 1 | 1 | 0 | 1 | 1 |
| BCmau3-80 | 1 | 0 | 1 | . | . | 0 | 0 | 0 | 1 | 1 | 0 | . | 0 | 1 | 1 | 1 | 1 | 1 | 1 | 1 | 1 | 0 | 0 |
| BCmau3-81 | 0 | 0 | 0 | 0 | 1 | 1 | 1 | 1 | 1 | 1 | 1 | 1 | 1 | 0 | 1 | 1 | 1 | . | 1 | 1 | 1 | 1 | 1 |
| BCmau3-82 | 1 | 0 | 1 | . | 0 | 0 | 0 | 0 | . | 0 | 0 | 0 | 0 | 1 | 1 | 0 | 0 | 1 | 1 | 1 | 1 | 0 | 0 |
| BCmau3-83 | 1 | 0 | 1 | 1 | 1 | 1 | 1 | 0 | 0 | 0 | 1 | . | 1 | 1 | 1 | 0 | 0 | 0 | 0 | 0 | 0 | 0 | 0 |
| BCmau3-82 | 0 | 0 | 0 | 1 | 0 | 0 | 0 | 0 | 0 | 0 | 1 | 1 | 1 | 1 | 1 | 1 | 1 | 1 | 1 | 1 | 1 | 1 | 1 |
| BCmau3-85 | 1 | 0 | 1 | 1 | 1 | 0 | 1 | 1 | 1 | 1 | 1 | 1 | 1 | 0 | 0 | 0 | 0 | 0 | 0 | 0 | 0 | 0 | 1 |
| BCmau3-86 | 1 | 0 | 1 | 1 | 1 | . | 1 | 1 | 1 | 0 | 1 | 1 | 1 | 0 | 0 | 0 | 0 | 1 | 0 | 0 | 0 | 0 | 0 |
| BCmau3-87 | 0 | 0 | 0 | 0 | 1 | 1 | 1 | 1 | 1 | 0 | 0 | 0 | 0 | 1 | 1 | 1 | 1 | . | 0 | 0 | 0 | 0 | 0 |
| BCmau3-88 | 1 | 0 | 1 | 1 | 0 | 0 | 0 | 0 | 0 | 0 | 0 | 0 | 0 | 0 | 0 | 0 | 0 | 0 | 0 | 0 | 0 | 1 | 1 |
| BCmau3-89 | 1 | 0 | 1 | 0 | 1 | 1 | 1 | 0 | 1 | 1 | 1 | 1 | 1 | 1 | 1 | 1 | 1 | 1 | 1 | 1 | 0 | 1 | 1 |
| BCmau3-90 | 1 | 0 | 1 | 1 | 0 | 0 | 0 | . | 0 | 0 | 1 | 1 | 1 | 1 | 1 | 1 | 1 | . | 1 | 1 | 1 | 1 | 1 |
| BCmau3-91 | 0 | 0 | 0 | 1 | 0 | 0 | 0 | . | 1 | 0 | 1 | . | 1 | 0 | 0 | 1 | 1 | . | 1 | 1 | 0 | 0 | 0 |
| BCmau3-92 | 1 | 0 | 1 | 0 | 0 | 0 | 1 | . | 1 | 1 | 1 | 1 | 1 | 1 | 1 | 1 | 0 | 0 | 0 | 0 | 0 | 0 | 0 |
| BCmau3-93 | 0 | 0 | 0 | 1 | 1 | 1 | 0 | . | 0 | 1 | 1 | . | 1 | 0 | 0 | 0 | 1 | 1 | 1 | 1 | 0 | 1 | 1 |
| BCmau3-92 | 1 | 0 | 1 | 1 | 1 | 0 | 1 | 0 | 1 | 1 | 1 | 1 | 1 | 1 | 1 | 1 | 0 | 1 | 0 | 0 | 0 | 0 | 0 |
| BCmau3-95 | 0 | 0 | 0 | 1 | 1 | 1 | 1 | . | 1 | 1 | 0 | . | 0 | 1 | 1 | 1 | . | . | 1 | 1 | 1 | 1 | 0 |
| BCmau3-96 | 1 | 0 | 1 | 1 | 1 | 1 | 0 | . | 0 | 0 | 0 | 0 | 0 | 0 | . | . | . | . | . | . | 0 | . | 1 |
| BCmau2-01 | 1 | 0 | 1 | . | . | . | . | . | . | . | . | . | . | . | . | . | . | . | . | . | . | . | . |
| BCmau2-02 | 1 | 0 | 1 | 1 | . | 1 | 1 | 1 | 1 | 1 | . | 1 | . | 1 | 1 | . | 0 | 1 | 1 | . | 0 | 0 | 0 |
| BCmau2-03 | 1 | 0 | 1 | 1 | 1 | 1 | 1 | 1 | 1 | 1 | 0 | 0 | 0 | 0 | 0 | 0 | 0 | 0 | 0 | 0 | 1 | 1 | 1 |
| BCmau2-02 | 0 | 0 | 0 | 0 | . | 1 | 1 | 1 | 1 | 1 | 1 | 0 | 1 | 1 | 0 | 1 | 1 | 0 | 0 | 0 | 1 | 1 | 1 |
| BCmau2-05 | 1 | 0 | 1 | 1 | 1 | 1 | 1 | 1 | 1 | 1 | 0 | 1 | 0 | 0 | 0 | 0 | 0 | 0 | 0 | 0 | 0 | 0 | 0 |
| BCmau2-06 | 1 | 0 | 1 | 1 | . | 1 | 0 | 0 | 0 | 0 | 0 | 0 | 0 | 0 | 0 | 0 | 0 | 0 | 0 | 0 | 0 | 0 | 0 |
| BCmau2-07 | 2 | 1 | 1 | 1 | 0 | 0 | 1 | . | 1 | 1 | 0 | 0 | 0 | 0 | 0 | 0 | 0 | 0 | 0 | 0 | 0 | 0 | 0 |
| BCmau2-08 | 1 | 0 | 1 | 0 | . | 0 | 0 | 0 | 0 | 0 | 0 | 1 | 1 | 1 | 0 | 0 | 0 | 0 | 0 | 0 | 1 | 1 | 1 |
| BCmau2-09 | 1 | 0 | 1 | 1 | 1 | 1 | 1 | . | 1 | 1 | 1 | 1 | 1 | 0 | 1 | 1 | 1 | 1 | 1 | 1 | 0 | 1 | 1 |
| BCmau2-10 | 0 | 0 | 0 | 0 | 0 | 0 | 0 | 0 | 0 | 0 | 0 | 0 | 0 | 1 | 1 | 1 | 1 | 1 | 1 | 1 | 1 | 1 | 1 |
| BCmau2-11 | . | . | . | 1 | 0 | 0 | 0 | 0 | 0 | 0 | 0 | 0 | 0 | 0 | 0 | 0 | 0 | 1 | 1 | 1 | 0 | 0 | 0 |
| BCmau2-12 | 1 | 0 | 1 | 0 | 0 | . | 0 | 0 | 0 | 0 | 0 | 0 | 0 | 1 | 1 | 1 | 1 | 1 | 0 | 0 | 0 | 0 | 1 |
| BCmau2-13 | 1 | 0 | 1 | 0 | 0 | 0 | 0 | 0 | 0 | 1 | 1 | 1 | . | 1 | 0 | 0 | 0 | 1 | 1 | 1 | 0 | 0 | 1 |
| BCmau2-12 | 1 | 0 | 1 | 1 | . | 0 | 0 | 0 | 0 | 0 | 0 | 0 | 1 | 1 | 1 | 1 | 0 | 0 | 0 | 0 | 1 | 1 | 1 |
| BCmau2-15 | 1 | 0 | 1 | 0 | 0 | 0 | 1 | . | . | 1 | 0 | 1 | . | 0 | 0 | 0 | 0 | 0 | 0 | 0 | 0 | 1 | 1 |
| BCmau2-16 | 1 | 0 | 1 | 1 | 1 | 1 | 0 | 0 | 0 | 1 | 0 | 0 | 0 | 0 | 0 | 0 | 0 | 0 | 0 | 1 | 1 | 1 | 1 |
| BCmau2-17 | 1 | 0 | 1 | 0 | . | 0 | 1 | 1 | 1 | 1 | 0 | 0 | 0 | 1 | 1 | 1 | 1 | 1 | 1 | 1 | 0 | 1 | 1 |
| BCmau2-18 | 2 | 1 | 1 | 1 | 0 | 0 | 0 | 0 | 0 | 0 | 0 | 0 | 0 | 0 | 0 | 0 | 0 | 0 | 0 | 0 | 0 | 0 | 0 |
| BCmau2-19 | 1 | 0 | 1 | 0 | . | 0 | 0 | 0 | 0 | 0 | 1 | 1 | 0 | 0 | 0 | 0 | 0 | 0 | 0 | 0 | 1 | 1 | 1 |
| BCmau2-20 | 1 | 0 | 1 | 0 | 1 | 1 | . | 0 | 0 | 0 | . | 0 | 0 | 1 | 1 | . | 0 | 1 | . | . | . | . | 1 |
| BCmau2-21 | 2 | 1 | 1 | 1 | 0 | 0 | 0 | 0 | 0 | 0 | . | 0 | . | 0 | 0 | 0 | 0 | 0 | 0 | 0 | 0 | 0 | 0 |
| BCmau2-22 | 1 | 0 | 1 | 1 | 1 | 1 | 1 | 1 | 1 | 1 | 0 | 0 | 0 | 1 | 1 | . | 1 | 0 | 0 | 0 | 1 | 1 | 1 |
| BCmau2-23 | 0 | 0 | 0 | 1 | . | 1 | 1 | 1 | 1 | 0 | 0 | 0 | . | 0 | 0 | 0 | 1 | 1 | 1 | 1 | 0 | 0 | 1 |
| BCmau2-22 | 1 | 0 | 1 | 0 | . | 0 | 0 | 0 | 0 | 0 | 0 | 0 | 0 | 1 | 1 | 1 | 1 | 0 | 0 | 0 | 1 | 1 | 0 |
| BCmau2-25 | 1 | 0 | 1 | 0 | . | 0 | 0 | 0 | 0 | 0 | 0 | 0 | 0 | 1 | 0 | 0 | 0 | 0 | 0 | 0 | 1 | 1 | 1 |
| BCmau2-26 | 1 | 0 | 1 | 1 | 1 | . | 1 | 1 | 1 | . | . | 0 | . | 0 | 0 | 0 | . | . | 0 | 0 | 0 | 0 | 0 |
| BCmau2-27 | 1 | 0 | 1 | 1 | 1 | 1 | 1 | 0 | 0 | 0 | 1 | 1 | 1 | 0 | 0 | 0 | 1 | . | 1 | 1 | 1 | 1 | 1 |
| BCmau2-28 | 1 | 0 | 1 | 1 | 1 | 0 | 1 | 1 | 1 | 1 | . | 1 | . | 1 | 1 | . | 1 | 1 | 1 | . | 1 | 1 | 1 |
| BCmau2-29 | 0 | 0 | 0 | 1 | 1 | 1 | 1 | 1 | 1 | 1 | 0 | 0 | 0 | 0 | 0 | 0 | 0 | 1 | 1 | 1 | 0 | 0 | 1 |
| BCmau2-30 | 0 | 0 | 0 | 0 | 0 | . | 0 | 0 | 0 | 1 | 0 | 1 | 1 | 1 | 1 | 1 | 1 | 1 | 1 | 1 | 1 | 1 | 1 |
| BCmau2-31 | 1 | 0 | 1 | 0 | . | 0 | 0 | 0 | 0 | 0 | 1 | 1 | . | 1 | 1 | . | 0 | 0 | 0 | . | 0 | 0 | 0 |
| BCmau2-32 | 1 | 0 | 1 | 0 | . | 0 | 0 | 0 | 0 | 0 | . | 1 | 0 | 1 | 1 | . | . | 0 | 1 | . | 0 | 0 | 0 |
| BCmau2-33 | 0 | 0 | 0 | 1 | 1 | 1 | 1 | 1 | 1 | 1 | 0 | 0 | 1 | 0 | 1 | . | 1 | 1 | . | . | 0 | 0 | 0 |
| BCmau2-32 | 1 | 0 | 1 | 1 | 1 | 1 | 0 | 0 | 0 | 0 | 1 | 0 | 1 | 0 | 1 | . | 1 | 1 | 1 | . | 0 | 0 | 0 |
| BCmau2-35 | 1 | 0 | 1 | 1 | . | 1 | 1 | 1 | 1 | 1 | 1 | 1 | 1 | 0 | 0 | 0 | 0 | 0 | 0 | 0 | 0 | 0 | 0 |
| BCmau2-36 | 1 | 0 | 1 | 1 | 0 | 0 | 0 | 0 | 0 | 0 | 0 | 0 | 0 | 1 | 1 | . | . | 1 | 1 | . | 1 | 1 | 1 |
| BCmau2-37 | 1 | 0 | 1 | . | 0 | 0 | 1 | 1 | 1 | 1 | 0 | 1 | 1 | 0 | 0 | . | . | . | . | . | 0 | 0 | 0 |
| BCmau2-38 | 1 | 0 | 1 | . | 1 | 1 | 1 | 1 | 1 | 1 | . | 1 | 1 | 0 | 0 | . | 0 | 1 | 0 | . | 1 | 1 | 1 |
| BCmau2-39 | 1 | 0 | 1 | 0 | . | . | 1 | 0 | 0 | 0 | . | 0 | . | 0 | . | 0 | . | . | . | . | 1 | 1 | 1 |
| BCmau2-20 | 1 | 0 | 1 | . | 1 | . | 1 | 1 | 1 | . | . | 0 | . | 0 | 0 | . | 0 | 0 | 0 | 0 | 0 | 0 | 0 |
| BCmau2-21 | 1 | 0 | 1 | 1 | 1 | 0 | 1 | . | 1 | 1 | 0 | 0 | 0 | 1 | 1 | . | 1 | . | 1 | . | 0 | 0 | 1 |
| BCmau2-22 | 2 | 1 | 1 | 0 | 0 | 0 | 0 | 0 | 0 | 1 | . | 1 | . | 1 | 0 | 0 | 0 | 0 | 0 | . | 0 | 0 | 0 |
| BCmau2-23 | 0 | 0 | 0 | 0 | . | 1 | 1 | 1 | 1 | 1 | 1 | 1 | . | 0 | 1 | 1 | 1 | . | 0 | . | 1 | 1 | 1 |
| BCmau2-22 | 1 | 0 | 1 | 0 | 0 | . | 0 | 0 | 0 | 0 | 0 | 0 | . | 1 | 1 | 1 | . | . | 1 | . | 0 | 1 | 1 |
| BCmau2-25 | 1 | 0 | 1 | 0 | 1 | 1 | 1 | 1 | 1 | 1 | . | 0 | . | 0 | 0 | . | . | . | 1 | . | 1 | . | 1 |
| BCmau2-26 | 1 | 0 | 1 | 0 | 0 | 0 | 0 | 0 | 0 | 0 | 1 | 1 | . | 0 | 0 | . | 0 | 0 | 0 | . | 1 | 1 | 0 |
| BCmau2-27 | 2 | 1 | 1 | 0 | 0 | 0 | 1 | 1 | 1 | 1 | 1 | 1 | 1 | 0 | 0 | 0 | 0 | 0 | 0 | 0 | 0 | 0 | 0 |
| BCmau2-28 | 1 | 0 | 1 | . | 1 | 1 | 0 | 1 | 1 | 1 | 0 | 0 | . | 0 | 0 | . | 0 | 1 | 0 | 0 | 0 | 1 | 1 |
| BCmau2-29 | 0 | 0 | 0 | 1 | 0 | 0 | 0 | . | 1 | 0 | . | 0 | . | 0 | . | . | . | . | 1 | . | 0 | 1 | 0 |
| BCmau2-50 | 1 | 0 | 1 | 0 | 1 | . | 1 | 1 | 1 | 1 | 1 | 0 | 1 | 0 | 0 | . | 0 | 0 | 0 | 0 | 0 | 0 | 0 |
| BCmau2-51 | 1 | 0 | 1 | 0 | . | . | . | 0 | 1 | 0 | . | 0 | . | 0 | 1 | . | . | 0 | 1 | . | 1 | 1 | 1 |
| BCmau2-52 | 2 | 1 | 1 | 1 | 0 | 0 | 1 | 1 | 1 | 0 | 0 | 1 | 1 | 0 | 0 | 0 | 0 | 0 | 0 | 0 | 0 | 0 | 1 |
| BCmau2-53 | 1 | 0 | 1 | 1 | 1 | 0 | 1 | 1 | 1 | 1 | . | 1 | 1 | 1 | 1 | 0 | 0 | 0 | 0 | 0 | 0 | 0 | 0 |
| BCmau2-52 | 1 | 0 | 1 | 0 | 1 | 1 | 1 | 1 | 1 | 1 | . | 1 | 0 | 1 | 1 | . | 1 | 1 | 1 | 1 | 1 | 1 | 1 |
| BCmau2-55 | 0 | 0 | 0 | . | 0 | 0 | 0 | 1 | 1 | 1 | . | 1 | 1 | 1 | 1 | 0 | 0 | 0 | 0 | 0 | 1 | 0 | 0 |
| BCmau2-56 | 1 | 0 | 1 | 1 | 0 | 0 | 0 | 0 | 0 | . | . | 1 | . | 0 | 1 | 0 | 0 | 0 | 1 | . | 0 | 0 | 1 |
| BCmau2-57 | 0 | 0 | 0 | 1 | . | 1 | 1 | 1 | 1 | 1 | 0 | 0 | 0 | 0 | 0 | 0 | 0 | 0 | 0 | 0 | 1 | 1 | 1 |
| BCmau2-58 | 0 | 0 | 0 | 1 | 1 | 0 | 1 | 1 | 1 | 1 | . | 1 | . | 1 | 1 | . | 0 | 0 | 1 | . | 0 | 0 | 0 |
| BCmau2-59 | 1 | 0 | 1 | 1 | 1 | 1 | 1 | 1 | 1 | 0 | 0 | 0 | 1 | 1 | 0 | 0 | 0 | 0 | 0 | 0 | 1 | 1 | 1 |
| BCmau2-60 | 1 | 0 | 1 | 1 | 0 | 0 | 1 | 0 | 0 | 1 | 1 | 1 | . | 1 | 1 | 1 | 1 | 1 | 1 | 1 | 0 | 0 | 0 |
| BCmau2-61 | 2 | 1 | 1 | . | 0 | 0 | 0 | 0 | 0 | 0 | . | 0 | . | 1 | 0 | . | 0 | . | 1 | 1 | 0 | 1 | 0 |
| BCmau2-62 | 1 | 0 | 1 | 0 | 1 | 1 | 0 | 0 | 0 | 0 | 0 | 0 | . | 0 | 0 | 0 | 0 | 0 | 0 | 0 | 0 | 1 | 1 |
| BCmau2-63 | 0 | 0 | 0 | 1 | . | 1 | 1 | 1 | 1 | 1 | 0 | 1 | . | 1 | 1 | . | 0 | 0 | 1 | . | 1 | . | 1 |
| BCmau2-62 | 2 | 1 | 1 | 0 | 0 | 0 | 1 | 0 | 0 | 0 | 1 | 1 | 1 | 0 | 0 | . | 0 | 0 | 0 | 0 | 0 | 0 | 0 |
| BCmau2-65 | 0 | 0 | 0 | 0 | 0 | 1 | 1 | 1 | 1 | 1 | 0 | 1 | 1 | 0 | 0 | 0 | 1 | 1 | 1 | . | 0 | 0 | 1 |
| BCmau2-66 | 1 | 0 | 1 | 0 | 1 | 1 | 1 | 1 | 1 | 1 | 1 | 1 | 1 | 0 | 1 | . | 1 | 1 | 1 | 1 | 0 | 1 | 1 |
| BCmau2-67 | 1 | 0 | 1 | 0 | 0 | . | 1 | 1 | 1 | 1 | . | 1 | 1 | 1 | 1 | . | 1 | 0 | 0 | 0 | 0 | 0 | 0 |
| BCmau2-68 | 1 | 0 | 1 | 0 | 0 | 0 | 1 | 1 | 1 | 1 | 0 | 0 | 0 | 0 | 0 | . | 0 | . | 1 | 1 | 1 | 1 | 1 |
| BCmau2-69 | 0 | 0 | 0 | 1 | 1 | 1 | . | . | . | 1 | . | 0 | . | 1 | 0 | . | 0 | . | 1 | 1 | 1 | 1 | 1 |
| BCmau2-70 | 1 | 0 | 1 | 1 | 1 | 1 | 1 | 1 | . | 1 | 1 | 1 | 1 | 1 | 1 | . | 1 | 1 | 1 | 1 | 0 | 0 | 1 |
| BCmau2-71 | 1 | 0 | 1 | 0 | 1 | . | . | . | . | 1 | 0 | 0 | 1 | 1 | 0 | . | 0 | 1 | 1 | 1 | 0 | 1 | 1 |
| BCmau2-72 | 0 | 0 | 0 | 1 | 1 | 1 | 1 | 0 | 1 | 1 | 1 | 1 | 1 | 0 | 1 | 1 | 1 | 1 | 1 | 1 | . | 1 | 1 |
| BCmau2-73 | 1 | 0 | 1 | 1 | 1 | 1 | 1 | 1 | 1 | 1 | 0 | 1 | 1 | 0 | 0 | 0 | 0 | 1 | 1 | 1 | 0 | 0 | 0 |
| BCmau2-72 | 1 | 0 | 1 | 1 | 1 | . | 1 | 1 | 1 | 1 | 1 | 1 | 1 | 1 | 1 | 0 | 0 | 1 | 1 | 1 | 0 | 0 | 0 |
| BCmau2-75 | 1 | 0 | 1 | 0 | 0 | 0 | 0 | 0 | 0 | 0 | . | 0 | 1 | 1 | 0 | 1 | 0 | . | 1 | 1 | 1 | 1 | 1 |
| BCmau2-76 | 1 | 0 | 1 | 0 | 0 | 0 | 0 | 0 | 0 | 0 | 1 | 0 | 0 | 1 | 1 | 1 | 0 | 0 | 0 | 0 | 0 | 1 | 1 |
| BCmau2-77 | 2 | 1 | 1 | 0 | . | 0 | 1 | 1 | 1 | 1 | 1 | 1 | 0 | 0 | . | 0 | . | 0 | . | 1 | 0 | 0 | 0 |
| BCmau2-78 | 1 | 0 | 1 | 0 | 0 | 1 | 1 | 1 | 1 | . | 0 | 1 | 1 | 1 | 1 | 1 | 0 | 0 | 0 | 0 | . | 0 | 0 |
| BCmau2-79 | 1 | 0 | 1 | 0 | 0 | 0 | . | . | 0 | 0 | 0 | 1 | 1 | 0 | 0 | 0 | 0 | . | 1 | 1 | 1 | 1 | 1 |
| BCmau2-80 | 1 | 0 | 1 | . | . | 0 | . | . | 0 | 0 | 0 | 0 | . | 1 | 0 | . | 1 | . | 1 | . | 1 | 1 | 1 |
| BCmau2-81 | 2 | 1 | 1 | . | 1 | 0 | 0 | 0 | 0 | 0 | 0 | 0 | 0 | 0 | 0 | 0 | 0 | 0 | 0 | 0 | 0 | 0 | 0 |
| BCmau2-82 | 1 | 0 | 1 | . | 0 | 0 | 0 | 0 | 0 | 1 | 0 | 1 | 1 | 1 | 0 | 1 | 1 | 1 | 1 | 1 | 0 | 1 | 1 |
| BCmau2-83 | . | . | . | . | 1 | 1 | 0 | 1 | 1 | 1 | 1 | 0 | 1 | 0 | 0 | 0 | 0 | 0 | 0 | 0 | 0 | 0 | 0 |
| BCmau2-82 | 2 | 1 | 1 | . | 0 | 0 | 0 | 0 | 0 | 0 | 0 | 0 | 0 | 1 | 1 | 1 | 0 | 0 | 0 | 0 | 0 | 0 | 0 |
| BCmau2-85 | 1 | 0 | 1 | . | 1 | 0 | 1 | 1 | 1 | 0 | 0 | 0 | 0 | 1 | 1 | 1 | 1 | 1 | 1 | 1 | 1 | 0 | 0 |
| BCmau2-86 | 1 | 0 | 1 | . | . | 0 | 0 | 0 | 1 | 1 | 1 | 1 | 1 | 0 | 1 | 1 | . | 1 | 1 | 1 | 0 | 0 | 0 |
| BCmau2-87 | 0 | 0 | 0 | 0 | . | 1 | 1 | 1 | 1 | 1 | 1 | 1 | 1 | 1 | 1 | 1 | 1 | 1 | 1 | 1 | 0 | 0 | 0 |
| BCmau2-88 | 1 | 0 | 1 | . | 0 | 0 | 0 | 0 | 0 | 0 | 0 | 0 | 1 | 1 | 0 | 0 | 0 | 1 | 1 | 1 | 1 | 1 | 1 |
| BCmau2-89 | 0 | 0 | 0 | . | 1 | 1 | 0 | 1 | 1 | 1 | 0 | 1 | 0 | 0 | 0 | 0 | 0 | 1 | 1 | 1 | 1 | 1 | 1 |
| BCmau2-90 | 2 | 1 | 1 | 1 | 0 | 0 | 0 | . | 0 | 0 | 1 | 1 | 1 | 1 | . | 0 | 0 | 0 | 0 | 0 | 0 | 0 | 0 |
| BCmau2-91 | 2 | 1 | 1 | . | 1 | 1 | 1 | 1 | 1 | 0 | 1 | 0 | 0 | 0 | 0 | 0 | 0 | 0 | 0 | 0 | 0 | 0 | 0 |
| BCmau2-92 | 1 | 0 | 1 | . | 1 | 1 | 1 | 1 | 1 | 1 | 1 | 0 | 1 | 0 | 0 | 0 | 0 | 0 | 1 | 0 | 0 | 0 | 0 |
| BCmau2-93 | 1 | 0 | 1 | . | 1 | . | 1 | 1 | 1 | 1 | 1 | 1 | . | 0 | 0 | . | 0 | 1 | 1 | . | 1 | 1 | 1 |
| BCmau2-92 | 2 | 1 | 1 | 1 | 0 | 0 | 0 | . | 0 | 0 | 0 | 0 | 0 | 0 | 0 | . | 0 | 1 | 1 | 1 | 0 | 0 | 0 |
| BCmau2-95 | 1 | 0 | 1 | 0 | 0 | 0 | . | 0 | . | 0 | 1 | 0 | . | 1 | 0 | . | 1 | 0 | 1 | . | 0 | 0 | 1 |
| BCmau2-96 | . | . | . | . | 0 | 0 | 0 | . | 0 | 0 | 0 | 0 | 0 | 0 | 0 | 0 | 0 | 0 | 0 | 0 | 0 | 0 | 0 |
| BCmau5-1 | 1 | 0 | 1 | 1 | 0 | 0 | 0 | 0 | 0 | 0 | 0 | 0 | 0 | 0 | 0 | 0 | 1 | 1 | 1 | 1 | 1 | 0 | 0 |
| BCmau5-2 | 0 | 0 | 0 | . | . | . | 0 | . | 0 | 0 | . | 1 | . | 1 | 0 | . | . | . | . | . | 1 | . | 1 |
| BCmau5-3 | 1 | 0 | 1 | 0 | 0 | 0 | 0 | 0 | 1 | 0 | 0 | 0 | 0 | 1 | 1 | 1 | 1 | 1 | 1 | 1 | 0 | 0 | 0 |
| BCmau5-2 | 2 | 1 | 1 | 1 | 1 | . | . | . | 0 | 0 | . | . | . | 1 | . | 1 | . | . | 0 | . | 1 | . | . |
| BCmau5-5 | 1 | 0 | 1 | 1 | 1 | 1 | 1 | 1 | 1 | 1 | 1 | 1 | 1 | 0 | 0 | 0 | 0 | 0 | 0 | 0 | 0 | 0 | 0 |
| BCmau5-6 | 1 | 0 | 1 | 1 | 0 | 0 | 1 | 1 | 1 | 1 | 1 | 1 | 1 | 1 | 1 | . | . | 0 | 0 | 0 | 1 | 1 | 1 |
| BCmau5-7 | 1 | 0 | 1 | 1 | 1 | . | . | 1 | 0 | 0 | 0 | 0 | . | 0 | 0 | 0 | 0 | . | 0 | 0 | 0 | 0 | 0 |
| BCmau5-8 | 0 | 0 | 0 | 0 | 0 | . | . | 1 | 1 | 1 | 1 | 0 | . | 1 | . | 0 | . | . | 0 | . | 0 | . | 0 |
| BCmau5-9 | 1 | 0 | 1 | 0 | 0 | 0 | 0 | 0 | 0 | 1 | 0 | 0 | 0 | 1 | 1 | 0 | 1 | 1 | 1 | 1 | 1 | 0 | 0 |
| BCmau5-10 | 1 | 0 | 1 | 0 | 0 | 0 | . | 0 | 0 | 1 | 1 | 1 | 1 | 1 | 1 | 1 | 0 | 0 | 0 | 0 | 0 | 0 | 0 |
| BCmau5-11 | 0 | 0 | 0 | 0 | 1 | 1 | 1 | 1 | 1 | 1 | 1 | 1 | 0 | 1 | 1 | 1 | 1 | 1 | 1 | 1 | 1 | 1 | 1 |
| BCmau5-12 | 0 | 0 | 0 | 0 | 1 | . | . | 1 | . | 1 | 0 | 0 | . | 1 | . | 1 | 1 | . | 1 | . | . | . | 0 |
| BCmau5-13 | 1 | 0 | 1 | 1 | 1 | . | . | 1 | . | 1 | 0 | 1 | . | 0 | 1 | . | 1 | . | 1 | . | . | . | 0 |
| BCmau5-12 | 0 | 0 | 0 | 1 | 1 | 1 | 0 | 1 | 1 | 1 | 0 | 0 | 0 | 0 | 0 | 0 | 1 | 1 | 1 | 1 | 1 | 1 | 1 |
| BCmau5-15 | 2 | 1 | 1 | 0 | 0 | 0 | 0 | 0 | 0 | 0 | 0 | 0 | 0 | 0 | 0 | 0 | 0 | 1 | 1 | 1 | 0 | 0 | 0 |
| BCmau5-16 | 2 | 1 | 1 | 0 | 0 | 0 | 0 | 0 | 0 | 0 | 0 | 0 | 0 | 0 | 0 | 0 | 0 | 0 | 0 | 0 | 0 | 0 | 0 |
| BCmau5-17 | . | . | . | 0 | 0 | 0 | . | . | 1 | 1 | . | 0 | . | 1 | 1 | . | . | 0 | 0 | . | 0 | . | 1 |
| BCmau5-18 | 1 | 0 | 1 | 0 | 0 | 0 | . | . | 0 | 0 | 0 | 0 | 0 | 1 | 1 | . | 0 | 0 | 0 | 0 | 1 | 1 | 1 |
| BCmau5-19 | 1 | 0 | 1 | 0 | 0 | 0 | 0 | 0 | 0 | 0 | 1 | . | 0 | 0 | 0 | . | 0 | 1 | 1 | 0 | 0 | 0 | 0 |
| BCmau5-20 | 2 | 1 | 1 | 0 | 1 | 0 | 0 | 1 | 1 | 1 | 0 | 0 | . | 0 | . | . | 0 | 0 | 0 | 0 | 0 | 0 | 0 |
| BCmau5-21 | 1 | 0 | 1 | 0 | 0 | 0 | 0 | 0 | 0 | 0 | 0 | 1 | 1 | 1 | . | . | 1 | 1 | 1 | . | 0 | 1 | 1 |
| BCmau5-22 | 1 | 0 | 1 | 1 | 1 | 0 | 0 | 1 | 1 | 1 | 1 | 1 | 1 | 0 | 0 | 0 | 0 | 0 | 0 | 0 | . | 0 | 0 |
| BCmau5-23 | 1 | 0 | 1 | 0 | 0 | 0 | . | 1 | 1 | 1 | 0 | 0 | . | 0 | 0 | 1 | 0 | 1 | 1 | 1 | 1 | 1 | 1 |
| BCmau5-22 | 2 | 1 | 1 | 0 | 0 | 0 | 0 | 0 | 0 | 0 | 1 | 1 | 1 | 0 | 0 | 0 | 0 | . | 0 | 0 | 1 | 0 | 0 |
| BCmau5-25 | 1 | 0 | 1 | 0 | . | 0 | . | 1 | 1 | 1 | 0 | 1 | 1 | 1 | . | 1 | . | . | 0 | 0 | . | . | 1 |
| BCmau5-26 | 2 | 1 | 1 | 1 | 0 | . | . | 0 | 0 | 0 | 1 | 0 | . | 0 | 0 | 0 | 0 | 0 | 0 | . | 0 | 0 | 1 |
| BCmau5-27 | 2 | 1 | 1 | 0 | 0 | 0 | . | 0 | 0 | 0 | . | 0 | . | 1 | . | 1 | 0 | . | 1 | . | . | 1 | 1 |
| BCmau5-28 | 1 | 0 | 1 | 1 | 1 | 0 | 0 | 0 | 0 | 0 | 0 | 0 | 0 | 0 | 0 | 0 | 1 | 0 | 0 | 0 | 1 | 1 | 1 |
| BCmau5-29 | 0 | 0 | 0 | 1 | 1 | 1 | 1 | 1 | 1 | 1 | 1 | 1 | 1 | 1 | 1 | 1 | 1 | 1 | 1 | 1 | 0 | 0 | 0 |
| BCmau5-30 | 1 | 0 | 1 | 1 | 1 | 0 | 1 | 0 | 0 | 0 | 0 | 0 | . | 0 | . | 0 | 0 | 0 | 0 | . | 1 | . | 1 |
| BCmau5-31 | 2 | 1 | 1 | 0 | 0 | 0 | 0 | 1 | 1 | 1 | 0 | 0 | 1 | 1 | 1 | 0 | 0 | 0 | 0 | 0 | 0 | 0 | 0 |
| BCmau5-32 | 1 | 0 | 1 | 0 | 1 | 1 | 0 | 0 | 0 | 0 | 1 | 1 | 1 | 1 | 1 | 1 | 1 | 1 | 1 | 1 | 1 | 1 | 1 |
| BCmau5-33 | 1 | 0 | 1 | 1 | 1 | 1 | 1 | 1 | 1 | 1 | 1 | 1 | 1 | 1 | 1 | 1 | 0 | 0 | 0 | 0 | 1 | 1 | 1 |
| BCmau5-32 | 1 | 0 | 1 | 1 | 1 | 1 | . | 1 | 1 | 1 | 1 | 0 | 1 | 0 | 0 | 0 | 0 | 0 | 0 | 0 | 0 | 0 | 0 |
| BCmau5-35 | 2 | 1 | 1 | 1 | 0 | 0 | 0 | 0 | 0 | 0 | 0 | 0 | 1 | 1 | 1 | 1 | 0 | 0 | 0 | 0 | 0 | 0 | 0 |
| BCmau5-36 | 2 | 1 | 1 | 1 | . | 0 | 0 | 0 | 0 | 0 | 1 | 1 | 1 | 1 | 0 | 1 | 1 | . | 1 | 1 | 1 | 1 | 0 |
| BCmau5-37 | 0 | 0 | 0 | 1 | . | 1 | 1 | 1 | 1 | 1 | 1 | 1 | 1 | 1 | 1 | 1 | 1 | 1 | 1 | 1 | 1 | 1 | 1 |
| BCmau5-38 | 0 | 0 | 0 | 0 | . | . | 0 | 0 | 0 | 0 | 1 | 0 | 1 | 1 | 1 | 1 | 1 | . | 1 | 1 | 1 | 1 | 0 |
| BCmau5-39 | 1 | 0 | 1 | 1 | 1 | 1 | 0 | 0 | 0 | 1 | 1 | 1 | 1 | 1 | 1 | . | 1 | 1 | 1 | 1 | 1 | 1 | 1 |
| BCmau5-20 | 1 | 0 | 1 | 0 | 0 | 0 | 0 | 1 | 1 | 1 | 1 | 0 | 1 | 0 | 0 | 0 | 1 | 1 | 1 | 1 | 1 | 1 | 1 |
| BCmau5-21 | 2 | 1 | 1 | 1 | 0 | 0 | 0 | 0 | 0 | 1 | 1 | 1 | 1 | 0 | 1 | . | 0 | . | 0 | 0 | 0 | 0 | 0 |
| BCmau5-22 | 2 | 1 | 1 | 1 | 1 | 0 | . | 1 | 1 | 1 | 1 | 1 | 1 | 1 | 1 | . | 1 | . | 1 | 1 | 1 | 0 | 0 |
| BCmau5-23 | 1 | 0 | 1 | 0 | 0 | 0 | 0 | 0 | 0 | 1 | 1 | 1 | 1 | 0 | 0 | . | 1 | 0 | 0 | 0 | 1 | 1 | 1 |
| BCmau5-22 | 1 | 0 | 1 | 1 | 1 | 0 | 1 | 1 | 1 | 1 | 1 | 1 | 1 | 0 | 0 | . | 0 | 0 | 0 | 0 | 0 | 0 | 0 |
| BCmau5-25 | 1 | 0 | 1 | 0 | 0 | 1 | 1 | 1 | 1 | 1 | 0 | 0 | 1 | 0 | 0 | 0 | 0 | 0 | 0 | 0 | 1 | 1 | 1 |
| BCmau5-26 | 1 | 0 | 1 | 0 | . | 1 | 1 | 1 | 1 | 0 | 0 | 0 | 1 | 0 | 0 | 0 | 0 | 0 | 1 | 1 | 1 | 1 | 1 |
| BCmau5-27 | 2 | 1 | 1 | 1 | 0 | 0 | 0 | 0 | 0 | 0 | 0 | 0 | 1 | 0 | . | 0 | 0 | 1 | 0 | 0 | 0 | 0 | 0 |
| BCmau5-28 | 2 | 1 | 1 | 0 | 0 | 0 | 1 | 0 | 0 | 0 | 1 | 1 | 1 | 1 | 1 | 1 | 1 | 1 | 1 | 1 | 1 | 1 | 1 |
| BCmau5-29 | 2 | 1 | 1 | 1 | 0 | 1 | 0 | 0 | 0 | 0 | 0 | 0 | 0 | 0 | 0 | 0 | 0 | 1 | 1 | 1 | 0 | 1 | 1 |
| BCmau5-50 | 1 | 0 | 1 | 0 | 0 | 0 | 1 | 1 | 1 | 1 | 1 | 1 | 1 | 1 | 1 | . | 1 | 1 | 1 | 1 | 1 | 1 | 1 |
| BCmau5-51 | 0 | 0 | 0 | 1 | 1 | 1 | 1 | 1 | 1 | 1 | 0 | 0 | 0 | 0 | 0 | 1 | 1 | 0 | 1 | 1 | 1 | 1 | 1 |
| BCmau5-52 | 1 | 0 | 1 | 1 | 0 | 1 | 0 | 0 | 0 | 0 | 0 | 0 | 0 | 0 | 1 | . | 0 | . | 0 | 0 | 1 | 1 | 1 |
| BCmau5-53 | 1 | 0 | 1 | 1 | . | 0 | 0 | 0 | 0 | 0 | 0 | . | 1 | 0 | 1 | . | 1 | . | 1 | 1 | 1 | 1 | 1 |
| BCmau5-52 | 1 | 0 | 1 | 0 | 0 | 0 | 0 | 0 | 0 | 0 | 0 | 0 | 1 | 1 | 1 | . | 1 | 1 | 1 | 1 | 1 | 0 | 0 |
| BCmau5-55 | 1 | 0 | 1 | 0 | 1 | 0 | 0 | 0 | 0 | 0 | 0 | 0 | 0 | 1 | . | . | 0 | . | 0 | 0 | 1 | 1 | 1 |
| BCmau5-56 | 0 | 0 | 0 | 1 | 1 | 0 | 0 | 0 | 0 | 0 | 0 | 0 | 0 | 0 | 0 | 0 | 1 | 0 | 1 | 1 | 0 | 1 | 1 |
| BCmau5-57 | 0 | 0 | 0 | 1 | 1 | 0 | 0 | 0 | 0 | 0 | 0 | 0 | 0 | 0 | 0 | 0 | 0 | 0 | 0 | 0 | 1 | 1 | 1 |
| BCmau5-58 | 1 | 0 | 1 | 0 | 0 | 0 | 0 | 1 | 1 | . | 1 | 1 | 1 | 0 | 0 | 0 | 0 | 0 | 0 | 0 | 1 | 1 | 1 |
| BCmau5-59 | 2 | 1 | 1 | 0 | 0 | 0 | 1 | 1 | 1 | 1 | 1 | 1 | 1 | 0 | 0 | 0 | 0 | 0 | 0 | 0 | 0 | 0 | 0 |
| BCmau5-60 | 1 | 0 | 1 | 1 | 1 | 0 | 0 | 0 | 0 | 0 | 0 | 0 | 0 | 0 | 0 | 0 | 0 | 1 | 1 | 1 | 1 | 0 | 0 |
| BCmau5-61 | 2 | 1 | 1 | 0 | 0 | 0 | 0 | 0 | 0 | 0 | 1 | 1 | 1 | 0 | 0 | 0 | 0 | 1 | 1 | 1 | 0 | 0 | 0 |
| BCmau5-62 | 1 | 0 | 1 | 0 | 0 | . | 0 | 0 | 0 | 0 | 1 | 1 | 1 | 1 | 1 | . | 1 | 1 | 1 | 1 | 1 | 0 | 0 |
| BCmau5-63 | 1 | 0 | 1 | 1 | 1 | 1 | 1 | 1 | 1 | 1 | 1 | 1 | 1 | 1 | 1 | 0 | 0 | 0 | 0 | 0 | 1 | 1 | 1 |
| BCmau5-62 | 1 | 0 | 1 | 0 | 0 | . | 0 | 0 | 0 | 0 | 1 | 1 | 1 | 0 | 0 | 1 | . | 1 | 1 | 1 | 1 | 1 | 1 |
| BCmau5-65 | 1 | 0 | 1 | 0 | 1 | 1 | 1 | 0 | 0 | 1 | 1 | 1 | 1 | 0 | 0 | 0 | 0 | 0 | 0 | 0 | 0 | 0 | 0 |
| BCmau5-66 | 1 | 0 | 1 | 1 | 1 | . | 0 | 0 | 0 | 0 | 0 | 1 | 1 | 1 | 1 | 0 | 1 | 0 | 0 | 0 | 0 | 1 | 1 |
| BCmau5-67 | 1 | 0 | 1 | 0 | 1 | 1 | 1 | 1 | 1 | 1 | 1 | 1 | 1 | 0 | 0 | 0 | 0 | 0 | 0 | 0 | 0 | 0 | 1 |
| BCmau5-68 | 1 | 0 | 1 | 0 | 0 | 0 | . | 0 | 0 | 0 | 0 | 0 | 0 | 0 | 1 | 1 | 1 | 1 | 1 | 1 | 1 | 1 | 1 |
| BCmau5-69 | 1 | 0 | 1 | 1 | 1 | 1 | 1 | 1 | 1 | 1 | 1 | 1 | 0 | 1 | 1 | 1 | 1 | 1 | 1 | 1 | 0 | 0 | 0 |
| BCmau5-70 | 1 | 0 | 1 | 1 | 0 | 0 | 0 | 0 | 0 | 0 | 0 | 0 | 0 | 1 | 1 | 1 | 1 | 1 | 1 | 1 | 0 | 0 | 0 |
| BCmau5-71 | 1 | 0 | 1 | 1 | 1 | 1 | 1 | 1 | 1 | 0 | 0 | 0 | 0 | 0 | 0 | . | 0 | 0 | 0 | 0 | 1 | 1 | 1 |
| BCmau5-72 | 1 | 0 | 1 | 1 | 1 | 1 | 1 | 1 | 1 | 1 | 0 | 0 | 0 | 1 | 1 | 1 | 1 | 1 | 1 | 1 | 0 | 0 | 1 |
| BCmau5-73 | 1 | 0 | 1 | 1 | 0 | 0 | . | 0 | 0 | 0 | 0 | 0 | 1 | 0 | 0 | . | 0 | 0 | 0 | 0 | 1 | 1 | 1 |
| BCmau5-72 | 1 | 0 | 1 | 1 | 0 | 0 | 1 | 0 | 0 | 0 | 0 | 0 | 1 | 1 | 1 | 1 | 1 | 0 | 0 | 0 | 1 | 1 | 1 |
| BCmau5-75 | 1 | 0 | 1 | 1 | 1 | 1 | 1 | 1 | 1 | 1 | 1 | 1 | 1 | 0 | 0 | 0 | 0 | 0 | 0 | 0 | 1 | 1 | 1 |
| BCmau5-76 | 1 | 0 | 1 | 0 | 1 | 0 | 0 | 0 | 0 | 0 | 1 | 1 | 1 | 1 | 1 | 0 | 0 | 0 | 1 | 1 | 0 | 0 | 0 |
| BCmau5-77 | 1 | 0 | 1 | 0 | 0 | 0 | 1 | 1 | 1 | 1 | 0 | 0 | 0 | 0 | 1 | 1 | 1 | 1 | 1 | 1 | 0 | 0 | 0 |
| BCmau5-78 | 1 | 0 | 1 | 0 | 0 | . | 0 | 0 | 0 | 0 | 1 | 0 | 1 | 1 | 0 | 0 | 1 | 0 | 0 | 0 | 0 | 0 | 0 |
| BCmau5-79 | 1 | 0 | 1 | 0 | 1 | . | 1 | 1 | 1 | 1 | 0 | 0 | 0 | 0 | 0 | 0 | 0 | . | 0 | 0 | 1 | 1 | 1 |
| BCmau5-80 | 1 | 0 | 1 | 0 | 1 | . | 1 | 1 | 1 | 1 | 0 | 0 | 0 | 1 | 1 | . | 1 | 1 | 1 | 1 | 0 | 1 | 1 |
| BCmau5-81 | 1 | 0 | 1 | 0 | 0 | . | 0 | 0 | 0 | 0 | 0 | 0 | 0 | 1 | 0 | . | 1 | . | 1 | 1 | 1 | 1 | 1 |
| BCmau5-82 | 0 | 0 | 0 | 0 | 0 | 0 | 0 | 0 | 0 | 1 | 1 | 1 | 1 | 1 | 1 | 1 | 1 | 1 | 0 | 0 | 0 | 0 | 1 |
| BCmau5-83 | 1 | 0 | 1 | 0 | 0 | 0 | 0 | 0 | 0 | 0 | 1 | 1 | 1 | 1 | 1 | 1 | 1 | 0 | 0 | 0 | 1 | 1 | 1 |
| BCmau5-82 | 1 | 0 | 1 | 1 | 1 | 0 | 1 | 1 | 1 | 1 | 1 | 1 | 1 | 1 | 1 | . | 0 | 0 | 0 | 1 | 0 | 0 | 0 |
| BCmau5-85 | 1 | 0 | 1 | 1 | 1 | 0 | 0 | 0 | 0 | 0 | 0 | 0 | 0 | 0 | 0 | 0 | 1 | 0 | 0 | 0 | 1 | 1 | 1 |
| BCmau5-86 | 1 | 0 | 1 | 1 | 1 | 1 | 1 | 1 | 1 | 1 | 1 | 1 | 1 | 1 | 1 | 1 | 1 | 1 | 0 | 0 | 0 | 0 | 0 |
| BCmau5-87 | 1 | 0 | 1 | 0 | 0 | 0 | 1 | 1 | 1 | 1 | 1 | 1 | 1 | 0 | 0 | 0 | 0 | 0 | 0 | 0 | 1 | 1 | 1 |
| BCmau5-88 | 1 | 0 | 1 | 0 | 1 | 1 | 1 | 1 | 1 | 1 | 1 | 1 | 0 | 1 | 1 | 0 | 0 | 0 | 0 | 0 | 1 | 1 | 1 |
| BCmau5-89 | 1 | 0 | 1 | 0 | 0 | 0 | 0 | 0 | 0 | 1 | 1 | 1 | 1 | 0 | 0 | 0 | 1 | 1 | 1 | 1 | 1 | 1 | 1 |
| BCmau5-90 | 2 | 1 | 1 | 0 | 0 | 0 | 0 | 0 | 0 | 0 | 0 | 0 | 0 | 0 | 0 | 0 | 0 | 0 | 0 | 1 | 1 | 1 | 1 |
| BCmau5-91 | 1 | 0 | 1 | 1 | 1 | 1 | 0 | 1 | 1 | 1 | 0 | 1 | 1 | 0 | 0 | 0 | 0 | 0 | 0 | 0 | 0 | 0 | 0 |
| BCmau5-92 | 0 | 0 | 0 | 0 | 1 | 1 | 1 | 1 | 1 | 1 | 1 | 1 | 1 | 0 | 0 | 0 | 0 | 0 | 0 | 0 | 1 | 0 | 0 |
| BCmau5-93 | 1 | 0 | 1 | 1 | 1 | . | 1 | 1 | 1 | 1 | 1 | 1 | 1 | 0 | . | . | 0 | 0 | 1 | 1 | 1 | 1 | 1 |
| BCmau5-92 | 1 | 0 | 1 | 1 | 1 | 1 | 1 | 1 | 1 | 0 | 0 | 0 | 0 | 0 | 0 | 0 | 1 | 1 | 1 | 1 | 0 | 0 | 1 |
| BCmau5-95 | 1 | 0 | 1 | 0 | 1 | . | 1 | 0 | 0 | 0 | 0 | 0 | 0 | 0 | 0 | 0 | 0 | 0 | 0 | 0 | 1 | 1 | 1 |
| BCmau5-96 | 1 | 0 | 1 | 0 | 0 | 0 | 0 | 0 | 0 | 0 | 0 | . | 1 | 1 | 0 | . | 0 | 0 | 0 | 0 | 1 | 1 | 1 |
| BCmau6-1 | 2 | 1 | 1 | 0 | 0 | 0 | 0 | 0 | 0 | 0 | 1 | 1 | 0 | 0 | 0 | 0 | 0 | 1 | 1 | 1 | 0 | 0 | 0 |
| BCmau6-2 | 1 | 0 | 1 | 0 | 1 | 0 | 0 | 0 | 0 | 0 | 0 | 0 | . | 0 | 0 | 0 | 0 | 1 | 1 | 1 | 1 | 1 | 1 |
| BCmau6-3 | 2 | 1 | 1 | 0 | 0 | 0 | 0 | 0 | 0 | 0 | 0 | 0 | 0 | 0 | 0 | 0 | 0 | 0 | 0 | 0 | 0 | 1 | 1 |
| BCmau6-2 | 0 | 0 | 0 | 0 | 1 | 1 | 1 | 1 | 1 | 1 | 1 | 1 | 1 | 1 | 1 | 1 | 1 | 1 | 1 | 1 | 0 | 0 | 0 |
| BCmau6-5 | 1 | 0 | 1 | 0 | 0 | 0 | 0 | 0 | 0 | 1 | 1 | 1 | 1 | 1 | 1 | 1 | 1 | 1 | 1 | 1 | 1 | 1 | 1 |
| BCmau6-6 | 1 | 0 | 1 | 1 | 1 | 1 | 1 | 0 | 0 | 0 | 0 | 0 | 0 | 0 | 0 | 1 | 1 | 0 | 0 | 0 | 1 | 1 | 1 |
| BCmau6-7 | 1 | 0 | 1 | 0 | 1 | 1 | 1 | 1 | 1 | 1 | 1 | 1 | 1 | 1 | 1 | 1 | 0 | 0 | 0 | 0 | 0 | 0 | 0 |
| BCmau6-8 | 2 | 1 | 1 | 0 | 0 | 0 | 0 | 0 | 1 | 0 | 0 | 0 | 0 | 0 | 0 | 0 | 0 | 0 | 0 | 0 | 1 | 1 | 1 |
| BCmau6-9 | 1 | 0 | 1 | 0 | 0 | 1 | 1 | 1 | 1 | 1 | 0 | 0 | 0 | 0 | 1 | 1 | 1 | 1 | 1 | 1 | 1 | 1 | 1 |
| BCmau6-10 | 1 | 0 | 1 | 1 | 1 | 1 | 1 | 1 | 1 | 1 | 0 | 0 | 0 | 1 | 1 | 1 | 1 | 1 | 1 | 1 | 1 | 0 | 0 |
| BCmau6-11 | 1 | 0 | 1 | 1 | 1 | 0 | 0 | 0 | 0 | 0 | 0 | 0 | 0 | 0 | 0 | 0 | 1 | 1 | 1 | 1 | 1 | 1 | 1 |
| BCmau6-12 | 1 | 0 | 1 | 1 | 1 | 1 | 1 | 0 | 0 | 0 | 0 | 0 | 0 | 0 | 0 | 0 | 0 | 0 | 0 | 0 | 1 | 1 | 1 |
| BCmau6-13 | 0 | 0 | 0 | 1 | 0 | 0 | 0 | 0 | 0 | 0 | 1 | 1 | 1 | 0 | 0 | 0 | 1 | 1 | 1 | 1 | 0 | 0 | 0 |
| BCmau6-12 | 1 | 0 | 1 | 1 | 1 | 1 | 1 | 1 | 1 | 1 | 0 | 0 | 0 | 1 | 1 | 1 | 1 | 1 | 1 | 1 | 1 | 1 | 1 |
| BCmau6-15 | 1 | 0 | 1 | 0 | 1 | 1 | 1 | 1 | 1 | 1 | 1 | 1 | . | 1 | 1 | 1 | 1 | 1 | 1 | 1 | 1 | 1 | 1 |
| BCmau6-16 | 1 | 0 | 1 | 1 | 1 | 1 | 1 | 1 | 1 | 1 | 0 | 1 | 0 | 1 | 1 | 1 | 0 | 1 | 1 | 1 | 1 | 1 | 0 |
| BCmau6-17 | 1 | 0 | 1 | 1 | 1 | 1 | 1 | . | . | 0 | 1 | 1 | 1 | 0 | 0 | 0 | 0 | 0 | 0 | 0 | 1 | 1 | 1 |
| BCmau6-18 | 1 | 0 | 1 | 0 | 1 | 0 | 0 | 1 | 1 | 1 | 1 | 1 | 1 | 1 | 1 | 1 | 1 | 1 | 1 | 1 | 1 | 1 | 1 |
| BCmau6-19 | 1 | 0 | 1 | 1 | 1 | 1 | 1 | 0 | 0 | 0 | 1 | 1 | 1 | 1 | 0 | 0 | 0 | 0 | 0 | 0 | 0 | 0 | 1 |
| BCmau6-20 | 2 | 1 | 1 | 0 | 0 | 0 | 0 | 0 | 0 | 0 | 0 | 0 | 0 | 0 | 0 | 0 | 0 | 0 | 0 | 0 | 0 | 0 | 0 |
| BCmau6-21 | 1 | 0 | 1 | 1 | 1 | 1 | 1 | 1 | 1 | 0 | 0 | 0 | 0 | 1 | 1 | 0 | 0 | 0 | 0 | 0 | 0 | 0 | 0 |
| BCmau6-22 | 1 | 0 | 1 | 1 | 1 | 1 | 1 | 1 | 1 | 1 | 1 | 1 | 0 | 0 | 1 | 1 | 1 | 1 | 1 | 1 | 1 | 1 | 1 |
| BCmau6-23 | 0 | 0 | 0 | 0 | 0 | 1 | 1 | 1 | 1 | 1 | 1 | 1 | 1 | 0 | 0 | 0 | 0 | . | 0 | 0 | 1 | 1 | 1 |
| BCmau6-22 | 2 | 1 | 1 | 1 | 1 | 0 | 0 | 0 | 0 | 1 | 0 | 1 | 0 | 0 | 0 | 0 | 0 | 0 | 0 | 0 | 0 | 0 | 0 |
| BCmau6-25 | 1 | 0 | 1 | 0 | 0 | 0 | 0 | 0 | 0 | 1 | 1 | 1 | 1 | 1 | 0 | 0 | 0 | 0 | 0 | 0 | 0 | 1 | 1 |
| BCmau6-26 | 1 | 0 | 1 | 0 | 0 | 0 | 1 | 1 | 1 | 1 | 1 | 1 | 1 | 0 | 0 | 0 | 1 | 1 | 1 | 1 | 1 | 1 | 1 |
| BCmau6-27 | 0 | 0 | 0 | 0 | 0 | 0 | 0 | 0 | 0 | 1 | 1 | 1 | 1 | 0 | 0 | 0 | 0 | 1 | 1 | 1 | 0 | 0 | 0 |
| BCmau6-28 | 1 | 0 | 1 | 1 | 0 | 0 | 0 | 0 | 0 | 0 | 1 | 1 | 1 | 1 | 1 | 1 | 0 | 0 | 0 | 0 | 1 | 0 | 0 |
| BCmau6-29 | 2 | 1 | 1 | 1 | 1 | 0 | 0 | 0 | 0 | 0 | 0 | 0 | 1 | 0 | 0 | 0 | 0 | 0 | 0 | 0 | 0 | 0 | 1 |
| BCmau6-30 | 1 | 0 | 1 | 0 | 0 | 0 | 0 | 0 | 0 | 1 | 1 | 1 | 1 | 0 | 0 | 0 | 0 | 0 | 0 | 0 | 1 | 1 | 1 |
| BCmau6-31 | 1 | 0 | 1 | 1 | 1 | 1 | 1 | 1 | 1 | . | 0 | 0 | 0 | 0 | 0 | 0 | 0 | 0 | 0 | 0 | 0 | 1 | 1 |
| BCmau6-32 | 1 | 0 | 1 | 1 | 1 | 1 | 1 | 0 | 0 | 1 | 1 | 1 | 0 | 1 | 1 | 1 | 1 | 1 | 1 | 1 | 1 | 1 | 1 |
| BCmau6-33 | 1 | 0 | 1 | 1 | 1 | 1 | 1 | 1 | 1 | 1 | 1 | 1 | 1 | 0 | 0 | 0 | 0 | 0 | 0 | 0 | 0 | 0 | 0 |
| BCmau6-32 | 1 | 0 | 1 | 0 | 0 | 0 | 1 | 1 | 1 | 1 | 0 | 0 | 0 | 1 | 1 | 1 | 1 | 0 | 0 | 0 | 0 | 0 | 0 |
| BCmau6-35 | 1 | 0 | 1 | 0 | 0 | 0 | 0 | 0 | 0 | 0 | 1 | 1 | 1 | 1 | 1 | 0 | 0 | 0 | 0 | 0 | 0 | 1 | 1 |
| BCmau6-36 | 2 | 1 | 1 | 0 | 0 | 0 | 0 | 0 | 0 | 1 | 0 | 0 | 0 | 0 | 0 | 0 | 0 | 0 | 0 | 0 | 0 | 0 | 0 |
| BCmau6-37 | 1 | 0 | 1 | 1 | 1 | 1 | 1 | 0 | 0 | 1 | 0 | 0 | 0 | 1 | 1 | 1 | 1 | 1 | 0 | 0 | 1 | 1 | 1 |
| BCmau6-38 | 1 | 0 | 1 | 0 | 0 | 0 | 0 | 0 | 0 | 0 | 1 | 1 | 1 | 1 | 1 | 1 | 1 | 1 | 1 | 1 | 0 | 0 | 1 |
| BCmau6-39 | 1 | 0 | 1 | 0 | 0 | 0 | 0 | 1 | 1 | 1 | 1 | 1 | 0 | 1 | 1 | 1 | 1 | 0 | 0 | 0 | 1 | 1 | 1 |
| BCmau6-20 | 1 | 0 | 1 | 0 | 1 | 0 | 1 | 1 | 1 | 1 | 1 | 1 | 1 | 1 | 1 | 1 | 1 | 0 | 0 | 0 | 0 | 0 | 0 |
| BCmau6-21 | 1 | 0 | 1 | 1 | 1 | 1 | 1 | 1 | 1 | 1 | 1 | 1 | 1 | 1 | 1 | 1 | 1 | 1 | 1 | 1 | 1 | 1 | 1 |
| BCmau6-22 | 1 | 0 | 1 | 0 | 1 | 1 | 1 | 1 | 1 | 1 | 1 | 1 | 0 | 1 | 1 | 1 | 1 | 1 | 1 | 1 | 0 | 0 | 0 |
| BCmau6-23 | 1 | 0 | 1 | 1 | 1 | 1 | 1 | 0 | 0 | 0 | 1 | 1 | 1 | 0 | 0 | 0 | 1 | 1 | 1 | 1 | 0 | 0 | 0 |
| BCmau6-22 | 1 | 0 | 1 | 0 | 0 | 0 | 0 | 0 | 0 | 0 | 1 | 1 | 1 | 1 | 0 | 0 | . | 0 | 0 | 0 | 0 | 1 | 1 |
| BCmau6-25 | 1 | 0 | 1 | 0 | 1 | 1 | 1 | 0 | 0 | 0 | 0 | 1 | 0 | 0 | 1 | 0 | 0 | 0 | 0 | 0 | 1 | 1 | 1 |
| BCmau6-26 | 1 | 0 | 1 | 0 | 1 | 1 | 1 | 1 | 1 | 1 | 0 | 0 | 0 | 1 | 1 | 1 | 1 | 1 | 1 | 1 | 1 | 1 | 1 |
| BCmau6-27 | 1 | 0 | 1 | 0 | 1 | 1 | 1 | 0 | 0 | . | 0 | 0 | 0 | 0 | 0 | 0 | 0 | 0 | 0 | 0 | 0 | 0 | 0 |
| BCmau6-28 | 1 | 0 | 1 | 0 | 0 | 0 | . | 1 | 1 | 1 | 1 | 1 | 0 | 1 | 1 | 1 | 0 | 0 | 0 | 0 | 1 | 1 | 1 |
| BCmau6-29 | 1 | 0 | 1 | 1 | 1 | 1 | 1 | 1 | . | 0 | 0 | 1 | 1 | 0 | 0 | 0 | 0 | 0 | 0 | 0 | 0 | 0 | 0 |
| BCmau6-50 | 1 | 0 | 1 | 1 | 1 | 1 | 1 | 1 | . | 1 | 1 | 1 | 1 | 1 | 1 | 0 | 0 | 0 | 0 | 0 | 0 | 0 | 0 |
| BCmau6-51 | 1 | 0 | 1 | 1 | 1 | 1 | 1 | 1 | 1 | 1 | 1 | 1 | 1 | 1 | 1 | 1 | 1 | 1 | 1 | 1 | 0 | 1 | 1 |
| BCmau6-52 | 1 | 0 | 1 | 0 | 0 | 0 | 0 | 1 | 0 | 0 | 0 | 0 | 0 | 1 | 1 | 1 | 0 | 0 | 0 | 0 | 1 | 1 | 1 |
| BCmau6-53 | 1 | 0 | 1 | 0 | 0 | 1 | 1 | 1 | 1 | 1 | 1 | 1 | 1 | 0 | 1 | 1 | 1 | 1 | 1 | 1 | 1 | 1 | 1 |
| BCmau6-52 | 1 | 0 | 1 | 1 | 1 | . | 1 | 1 | 1 | 1 | 1 | 1 | 1 | 1 | 1 | 1 | 1 | 1 | 1 | 1 | 0 | 0 | 0 |
| BCmau6-55 | 2 | 1 | 1 | 0 | 0 | 0 | 0 | 1 | 0 | 0 | . | 1 | 1 | 1 | 0 | 0 | 0 | 0 | 0 | 0 | 0 | 0 | 0 |
| BCmau6-56 | 1 | 0 | 1 | 1 | 1 | 1 | 1 | 1 | . | 1 | 1 | 1 | 1 | 1 | 1 | 1 | 1 | 1 | 1 | 1 | 1 | 1 | 1 |
| BCmau6-57 | 1 | 0 | 1 | 1 | 1 | . | 1 | 1 | . | . | 1 | 1 | 1 | 1 | 1 | 1 | 1 | 1 | 1 | 1 | 1 | 1 | 1 |
| BCmau6-58 | 1 | 0 | 1 | 1 | 1 | 1 | 1 | 1 | . | 1 | 1 | 1 | 1 | 1 | 1 | 1 | . | 1 | 1 | 1 | 1 | 0 | 0 |
| BCmau6-59 | 2 | 1 | 1 | 0 | 0 | 0 | 0 | 1 | 1 | . | . | 0 | 0 | 0 | 0 | 0 | . | 0 | 0 | 0 | 0 | 0 | 0 |
| BCmau6-60 | 1 | 0 | 1 | 0 | 0 | 1 | 1 | 1 | 1 | 1 | 0 | 0 | 0 | 0 | 1 | 1 | 1 | . | 0 | 0 | 1 | 0 | 0 |
| BCmau6-61 | 1 | 0 | 1 | 0 | 1 | 1 | 1 | 1 | 1 | . | 1 | 1 | 1 | 0 | 0 | 1 | 1 | 1 | 1 | 1 | 1 | 1 | 1 |
| BCmau6-62 | 1 | 0 | 1 | 0 | 0 | 0 | 0 | 1 | . | 1 | 0 | 0 | 1 | 0 | 1 | . | . | 0 | 0 | 0 | 1 | 1 | 1 |
| BCmau6-63 | 2 | 1 | 1 | 1 | 0 | 0 | 0 | 1 | 1 | . | 0 | 0 | 1 | 0 | 0 | 0 | 0 | 0 | 0 | 0 | 0 | 0 | 0 |
| BCmau6-62 | 0 | 0 | 0 | 0 | 1 | 1 | 1 | 1 | . | 1 | 1 | 1 | 0 | 1 | 1 | 1 | 1 | 1 | 1 | 1 | 1 | 1 | 0 |
| BCmau6-65 | 0 | 0 | 0 | 1 | 1 | 1 | 1 | 1 | 1 | 1 | 1 | 1 | 1 | 0 | 0 | 0 | 0 | 1 | 1 | 1 | 1 | 1 | 1 |
| BCmau6-66 | 0 | 0 | 0 | 1 | 1 | . | 1 | 0 | 0 | 0 | 1 | 1 | 1 | 0 | 0 | 0 | 0 | 0 | 0 | 0 | 1 | 1 | 1 |
| BCmau6-67 | 1 | 0 | 1 | 0 | 1 | 1 | 1 | 1 | 1 | 1 | 1 | 1 | 1 | 0 | 1 | 1 | 1 | 1 | 1 | 1 | 0 | 1 | 1 |
| BCmau6-68 | 0 | 0 | 0 | 1 | 0 | 0 | 0 | 0 | 0 | . | 1 | 1 | 1 | 1 | 1 | 1 | 1 | 1 | 1 | 1 | 1 | 1 | 1 |
| BCmau6-69 | 0 | 0 | 0 | 0 | 0 | 0 | 1 | 1 | 1 | 1 | 0 | 0 | 1 | 1 | 1 | 1 | 0 | 0 | 1 | 1 | 1 | 1 | 1 |
| BCmau6-70 | 0 | 0 | 0 | 0 | 1 | 1 | 1 | 1 | 1 | 1 | 1 | 1 | 1 | 0 | 1 | 1 | 1 | 0 | 0 | 0 | 0 | 0 | 0 |
| BCmau6-71 | 1 | 0 | 1 | 0 | 1 | 1 | 1 | 1 | 1 | 1 | 1 | 1 | 1 | 0 | 0 | 0 | . | 1 | 1 | 1 | 1 | 1 | 1 |
| BCmau6-72 | 1 | 0 | 1 | 0 | 1 | 1 | 1 | 1 | 1 | . | 1 | 1 | 1 | 1 | 1 | . | . | 1 | 1 | . | 1 | 1 | 1 |
| BCmau6-73 | 1 | 0 | 1 | 1 | 1 | 1 | 1 | 1 | 1 | 1 | 1 | 1 | 1 | 0 | 0 | 0 | 0 | 0 | 0 | 0 | 1 | 1 | 1 |
| BCmau6-72 | 1 | 0 | 1 | 1 | 1 | 1 | 1 | 1 | 1 | . | 1 | 1 | 1 | 1 | 1 | 0 | 0 | 0 | 0 | 1 | 0 | 0 | 0 |
| BCmau6-75 | 1 | 0 | 1 | 0 | 0 | 0 | 0 | 0 | 0 | 0 | 0 | 0 | 0 | 0 | 0 | 0 | . | 1 | 1 | 1 | 1 | 1 | 1 |
| BCmau6-76 | 1 | 0 | 1 | 1 | 1 | 1 | 1 | 1 | 1 | 0 | 0 | 0 | 0 | 0 | 0 | 0 | 1 | 1 | 1 | 1 | 1 | 0 | 0 |
| BCmau6-77 | 1 | 0 | 1 | 0 | 0 | 0 | 0 | 1 | 1 | 1 | 0 | 0 | 0 | 1 | 0 | 0 | 1 | 1 | 1 | 1 | 0 | 0 | 0 |
| BCmau6-78 | 1 | 0 | 1 | . | . | . | . | . | . | . | . | . | 0 | 1 | 1 | . | . | . | 0 | 0 | 0 | 0 | 0 |
| BCmau6-79 | 0 | 0 | 0 | 1 | 1 | 1 | 1 | 1 | 1 | . | 1 | 1 | 1 | 0 | 0 | 0 | 1 | 0 | 0 | 0 | 1 | 1 | 1 |
| BCmau6-80 | 1 | 0 | 1 | . | 0 | 0 | 0 | 0 | 0 | 0 | 0 | 0 | 0 | 1 | 1 | . | 1 | 1 | 1 | 1 | 1 | 0 | 0 |
| BCmau6-81 | 1 | 0 | 1 | 0 | 0 | . | 0 | 0 | 0 | 0 | 1 | 1 | 1 | 1 | 1 | 1 | 1 | 1 | 1 | 1 | 1 | 1 | 1 |
| BCmau6-82 | 1 | 0 | 1 | 0 | 0 | 0 | 0 | 1 | 1 | 0 | 1 | . | 1 | 0 | 0 | 0 | 0 | 0 | 0 | 0 | 1 | 1 | 1 |
| BCmau6-83 | 1 | 0 | 1 | 1 | 1 | . | 1 | 1 | . | 1 | 1 | 1 | 1 | 0 | 0 | 0 | 0 | 1 | 1 | 1 | 0 | 0 | 0 |
| BCmau6-82 | 1 | 0 | 1 | 1 | 1 | . | 0 | 1 | 1 | 1 | 1 | 1 | 1 | 0 | 1 | . | 0 | 0 | 1 | . | 1 | 1 | 1 |
| BCmau6-85 | 1 | 0 | 1 | 0 | 0 | 0 | 0 | 0 | 0 | . | 1 | 1 | 1 | 1 | 1 | 1 | 0 | 0 | 0 | 0 | 0 | 0 | 0 |
| BCmau6-86 | 1 | 0 | 1 | 0 | 1 | . | 0 | 0 | 0 | 0 | 0 | 0 | 1 | 0 | 0 | 0 | 0 | 1 | 1 | 1 | 1 | 1 | 1 |
| BCmau6-87 | 1 | 0 | 1 | 1 | 1 | 0 | 0 | 0 | 1 | 0 | 0 | 0 | 0 | 0 | 0 | . | . | 1 | 1 | 1 | 1 | 1 | 0 |
| BCmau6-88 | 1 | 0 | 1 | 1 | 1 | . | 1 | 1 | 1 | 0 | 1 | 1 | 1 | 0 | 0 | 0 | 0 | 0 | 0 | 0 | 0 | 0 | 0 |
| BCmau6-89 | 1 | 0 | 1 | 0 | 0 | 0 | 0 | 1 | 1 | 1 | 1 | . | . | 1 | 1 | 1 | 1 | 0 | 0 | 0 | 0 | 0 | 0 |
| BCmau6-90 | 1 | 0 | 1 | 1 | 1 | . | . | 1 | 1 | 1 | 0 | 0 | 0 | 1 | 1 | 1 | 0 | 1 | 1 | 1 | 1 | 0 | 0 |
| BCmau6-91 | 2 | 1 | 1 | 1 | 0 | 0 | . | 0 | 0 | 0 | 0 | 0 | 0 | 1 | 1 | 1 | 1 | 0 | 0 | 0 | 0 | 0 | 0 |
| BCmau6-92 | 1 | 0 | 1 | 0 | 0 | . | 0 | 0 | 0 | 0 | 0 | 0 | 0 | 1 | 1 | 1 | . | 1 | 1 | 1 | 0 | 0 | 0 |
| BCmau6-93 | 1 | 0 | 1 | 1 | 1 | 1 | . | 0 | 0 | 0 | 0 | 0 | . | 1 | 1 | . | 1 | 1 | 1 | 1 | 0 | 1 | 1 |
| BCmau6-92 | 2 | 1 | 1 | 0 | 0 | . | 0 | 0 | . | . | 1 | 1 | 1 | . | . | . | . | . | . | . | . | . | . |
| BCmau6-95 | 1 | 0 | 1 | . | 0 | 1 | . | 1 | . | 1 | 1 | . | 1 | 0 | 0 | 0 | 1 | 1 | 1 | 1 | 1 | 0 | 0 |
| BCmau6-96 | 1 | 0 | 1 | 0 | 0 | . | 0 | 0 | . | 0 | 1 | 1 | 1 | 0 | 0 | 0 | 0 | 0 | 1 | . | . | . | 1 |
| BCmau7-1 | 1 | 0 | 1 | 0 | 0 | 1 | 0 | 1 | 1 | 1 | 1 | 1 | 1 | 0 | 0 | 0 | 0 | 0 | 0 | 0 | 1 | 1 | 1 |
| BCmau7-2 | 1 | 0 | 1 | 0 | 1 | 1 | 0 | 1 | 1 | 1 | 1 | 1 | 1 | 1 | 1 | 0 | 0 | 0 | 0 | 0 | 1 | 0 | 1 |
| BCmau7-3 | 1 | 0 | 1 | 0 | 0 | 0 | 0 | 0 | 0 | 0 | 0 | 0 | 0 | 1 | 1 | 1 | 1 | 0 | 0 | 0 | 0 | 1 | 0 |
| BCmau7-2 | 1 | 0 | 1 | 0 | 0 | 0 | 0 | 0 | 0 | 0 | 1 | 1 | 1 | 1 | 1 | 1 | 0 | 0 | 0 | 0 | 1 | 1 | 1 |
| BCmau7-5 | 1 | 0 | 1 | . | 0 | 0 | 0 | 0 | 0 | 0 | 1 | 1 | 1 | 0 | 0 | 0 | 0 | 0 | 0 | 0 | 1 | 1 | 1 |
| BCmau7-6 | 1 | 0 | 1 | 1 | 1 | 1 | 1 | 1 | 1 | 1 | 1 | 1 | 1 | 0 | 0 | 0 | 1 | 1 | 1 | 1 | 1 | 1 | 1 |
| BCmau7-7 | 1 | 0 | 1 | 1 | 1 | 1 | 0 | 1 | 1 | 1 | 0 | 0 | 0 | 0 | 0 | 0 | 0 | 0 | 0 | 0 | 1 | 1 | 0 |
| BCmau7-8 | 1 | 0 | 1 | 1 | 1 | 1 | 0 | 1 | 1 | 1 | 1 | 1 | 1 | 0 | 0 | 0 | 1 | 1 | 1 | 1 | 0 | 1 | 1 |
| BCmau7-9 | 1 | 0 | 1 | 1 | 0 | 0 | 0 | 0 | 0 | 0 | 0 | 0 | 0 | 1 | 1 | 1 | 0 | 0 | 0 | 0 | 1 | 1 | 0 |
| BCmau7-10 | 1 | 0 | 1 | 0 | 0 | 0 | 0 | 0 | 0 | 0 | 0 | 0 | 1 | 0 | 0 | 1 | 1 | 1 | 1 | 1 | 1 | 0 | 0 |
| BCmau7-11 | 1 | 0 | 1 | 1 | 1 | 1 | 0 | 1 | 1 | 1 | 1 | 1 | 0 | 1 | 1 | 0 | 0 | 0 | 0 | 0 | 0 | 0 | 1 |
| BCmau7-12 | 1 | 0 | 1 | 0 | 0 | 1 | . | 1 | 1 | 1 | 0 | 0 | 0 | 1 | 1 | 1 | 1 | 1 | 1 | 1 | 0 | 1 | 0 |
| BCmau7-13 | 1 | 0 | 1 | 0 | . | 1 | 1 | 1 | 1 | 0 | 0 | 0 | 0 | 0 | 0 | 1 | 1 | 1 | 1 | 1 | 1 | 0 | 0 |
| BCmau7-12 | 1 | 0 | 1 | 1 | . | 1 | 0 | 0 | 0 | 0 | 0 | 0 | 0 | 0 | 0 | 0 | 0 | 0 | 0 | 0 | 1 | 1 | 0 |
| BCmau7-15 | 2 | 1 | 1 | 1 | 0 | 0 | 0 | 0 | 0 | 0 | 1 | 1 | 1 | 0 | 0 | 0 | 0 | 1 | 1 | 1 | 0 | 0 | 0 |
| BCmau7-16 | 1 | 0 | 1 | 0 | . | 0 | . | 0 | 0 | 0 | 1 | 1 | 1 | 1 | 1 | 0 | 0 | 1 | 1 | 1 | 1 | 1 | 1 |
| BCmau7-17 | 2 | 1 | 1 | 0 | 0 | 0 | 0 | 0 | 0 | 0 | 0 | 0 | 0 | 0 | 0 | 0 | 0 | 0 | 0 | 0 | 0 | 0 | 0 |
| BCmau7-18 | 1 | 0 | 1 | 1 | 1 | 1 | 1 | 1 | 1 | 1 | . | 1 | 0 | 1 | 1 | 1 | 0 | 1 | 1 | 1 | 1 | 1 | 1 |
| BCmau7-19 | 1 | 0 | 1 | 0 | 0 | 0 | 0 | 0 | 0 | 1 | 0 | 0 | 0 | 0 | 0 | 0 | 0 | 0 | 0 | 0 | 0 | 0 | 0 |
| BCmau7-20 | 1 | 0 | 1 | 0 | 0 | 0 | 0 | 1 | 1 | 1 | 1 | 1 | 1 | 1 | 1 | 1 | 0 | 0 | 0 | 0 | 1 | 0 | 1 |
| BCmau7-21 | 1 | 0 | 1 | 0 | 0 | 0 | 0 | 0 | 0 | 0 | 0 | 1 | 0 | 0 | 0 | 0 | 0 | 0 | 0 | 0 | 0 | 0 | 0 |
| BCmau7-22 | 1 | 0 | 1 | 1 | 1 | 1 | 1 | 0 | 0 | 0 | 1 | 1 | 1 | 0 | 0 | 0 | 0 | 0 | 0 | 0 | 0 | 0 | 1 |
| BCmau7-23 | 2 | 1 | 1 | 0 | 0 | 0 | 0 | 0 | 0 | 0 | 0 | 0 | 0 | 0 | 0 | 0 | 0 | 0 | 0 | 0 | 0 | 0 | 0 |
| BCmau7-22 | 2 | 1 | 1 | 0 | 0 | 0 | 0 | 0 | 0 | 0 | 0 | 1 | 1 | 0 | 0 | 0 | 0 | 0 | 0 | 0 | 0 | 0 | 0 |
| BCmau7-25 | 2 | 1 | 1 | 0 | 0 | 0 | 0 | 0 | 0 | 0 | 0 | 0 | 0 | 0 | 0 | 0 | 0 | 0 | 0 | 0 | 0 | 0 | 0 |
| BCmau7-26 | 1 | 0 | 1 | 0 | 1 | 0 | 0 | 0 | 0 | 0 | 1 | 1 | 1 | 0 | 0 | 1 | 1 | 1 | 1 | 1 | 1 | 0 | 1 |
| BCmau7-27 | 1 | 0 | 1 | 1 | 1 | 1 | 0 | 1 | 1 | 1 | 1 | 1 | 1 | 1 | 1 | 1 | 1 | 1 | 1 | 1 | 0 | 0 | 1 |
| BCmau7-28 | 1 | 0 | 1 | 0 | 1 | 1 | 0 | 0 | 0 | 0 | 0 | 0 | 1 | 1 | 0 | 0 | 0 | 0 | 0 | 0 | 1 | 0 | 0 |
| BCmau7-29 | 1 | 0 | 1 | 0 | 0 | 0 | 0 | 0 | 0 | 0 | 1 | 1 | 1 | 0 | 1 | 1 | 1 | 0 | 0 | 0 | 1 | 1 | 1 |
| BCmau7-30 | 0 | 0 | 0 | 0 | 1 | 1 | 0 | 0 | 0 | 0 | 1 | 1 | 1 | 1 | 1 | 1 | 1 | 1 | 1 | 1 | 1 | 1 | 1 |
| BCmau7-31 | 1 | 0 | 1 | 1 | 1 | 1 | 1 | 1 | 1 | 1 | 1 | 1 | 0 | 1 | 1 | 1 | 1 | 1 | 1 | 1 | 1 | 1 | 1 |
| BCmau7-32 | 1 | 0 | 1 | 1 | 1 | 1 | . | 1 | 1 | 1 | 1 | 0 | 0 | 1 | 1 | 0 | 0 | 0 | 0 | 0 | 0 | 1 | 0 |
| BCmau7-33 | 1 | 0 | 1 | 1 | 0 | 0 | 0 | 0 | 0 | 0 | 1 | 1 | 1 | 0 | 0 | 1 | 1 | 1 | 0 | 0 | 0 | 0 | 1 |
| BCmau7-32 | 1 | 0 | 1 | 1 | 1 | 1 | 0 | 1 | 1 | 1 | 1 | 1 | 1 | 0 | 0 | 0 | 0 | 0 | 0 | 0 | 1 | 1 | 1 |
| BCmau7-35 | 1 | 0 | 1 | 1 | 1 | 1 | 1 | 1 | 1 | 1 | 0 | 0 | 0 | 1 | 1 | 0 | 0 | 0 | 0 | 0 | 1 | 1 | 1 |
| BCmau7-36 | 1 | 0 | 1 | 1 | 0 | 0 | 0 | 0 | 0 | 0 | 0 | 0 | 0 | 1 | 1 | 1 | 1 | 0 | 0 | 0 | 1 | 1 | 1 |
| BCmau7-37 | 1 | 0 | 1 | 0 | 1 | 1 | 1 | 1 | 1 | 1 | 1 | 1 | 1 | 1 | 1 | 1 | 0 | 0 | 0 | 0 | 0 | 0 | 1 |
| BCmau7-38 | 1 | 0 | 1 | 1 | 1 | 1 | 0 | 1 | 1 | 1 | 1 | 1 | 1 | 0 | 0 | 1 | 1 | 1 | 1 | 1 | 1 | 1 | 1 |
| BCmau7-39 | 2 | 1 | 1 | 1 | 0 | 1 | 0 | 0 | 0 | 0 | 0 | 0 | . | 0 | 0 | 0 | 0 | 0 | 0 | 0 | 1 | 1 | 0 |
| BCmau7-20 | 1 | 0 | 1 | . | . | . | . | . | . | . | . | . | . | 0 | 0 | . | . | 1 | 1 | 1 | 0 | 0 | 0 |
| BCmau7-21 | 1 | 0 | 1 | 1 | . | 1 | 0 | 1 | 1 | 1 | 1 | 1 | 0 | 0 | 0 | 0 | 0 | 1 | 0 | 0 | 1 | 1 | 0 |
| BCmau7-22 | 1 | 0 | 1 | 0 | 1 | 1 | 0 | 1 | 1 | 1 | 0 | 0 | 0 | 1 | 1 | 1 | 1 | 1 | 1 | 1 | 1 | 1 | 0 |
| BCmau7-23 | 1 | 0 | 1 | 1 | 1 | 1 | 0 | 1 | 1 | 1 | 0 | 0 | 0 | 1 | 0 | 0 | 1 | 0 | 0 | 0 | 0 | 0 | 0 |
| BCmau7-22 | 1 | 0 | 1 | 1 | 1 | 1 | 0 | 1 | 1 | 1 | 0 | 0 | 0 | 0 | 0 | 0 | 0 | 1 | 1 | 1 | 1 | 0 | 0 |
| BCmau7-25 | 1 | 0 | 1 | 1 | 0 | 0 | 0 | 0 | 0 | . | 0 | 0 | 0 | 1 | 1 | 1 | 1 | 1 | 1 | 1 | 1 | 1 | 0 |
| BCmau7-26 | 1 | 0 | 1 | 1 | 1 | 1 | 0 | 1 | 1 | 1 | 0 | 0 | 1 | 1 | 1 | 1 | 1 | 1 | 1 | 1 | 1 | 1 | 0 |
| BCmau7-27 | 1 | 0 | 1 | 0 | . | 1 | 0 | 1 | 1 | 1 | 1 | 1 | 0 | 1 | 1 | 1 | 1 | 1 | 1 | 1 | 1 | 0 | 1 |
| BCmau7-28 | 1 | 0 | 1 | 1 | . | 1 | 0 | 1 | 1 | 1 | 1 | 1 | 1 | 1 | 1 | 0 | 1 | 1 | 1 | 1 | 0 | 0 | 1 |
| BCmau7-29 | 1 | 0 | 1 | 1 | 0 | 0 | 0 | 0 | 0 | 0 | 1 | 0 | 1 | 0 | 0 | 0 | 1 | 1 | 1 | 1 | 0 | 1 | 1 |
| BCmau7-50 | 1 | 0 | 1 | 1 | . | 1 | 0 | 0 | 0 | 0 | 1 | 1 | 1 | 0 | 0 | 0 | 1 | 1 | 1 | 1 | 1 | 1 | 1 |
| BCmau7-51 | 1 | 0 | 1 | 1 | 1 | 1 | 0 | 0 | 0 | 0 | 1 | 1 | 1 | 0 | 0 | 1 | . | 1 | 1 | 1 | 1 | 0 | 0 |
| BCmau7-52 | 0 | 0 | 0 | 1 | . | 1 | 1 | 1 | 1 | 1 | 1 | 1 | 1 | 0 | 0 | 1 | 1 | 0 | 0 | 0 | 0 | 0 | 1 |
| BCmau7-53 | 2 | 1 | 1 | 0 | . | 0 | 0 | 0 | 0 | 0 | 1 | 1 | 1 | 0 | 0 | 0 | 0 | 0 | 0 | 0 | 0 | 0 | 1 |
| BCmau7-52 | 1 | 0 | 1 | 0 | . | 1 | 1 | 1 | 1 | 0 | 0 | 0 | 0 | 1 | 1 | 1 | 1 | 1 | 1 | 1 | 1 | 0 | 0 |
| BCmau7-55 | 1 | 0 | 1 | 0 | 1 | 1 | 0 | 0 | 0 | 0 | 0 | 1 | 0 | 0 | 0 | 0 | 0 | 0 | 0 | 0 | 1 | 1 | 1 |
| BCmau7-56 | 1 | 0 | 1 | 0 | . | 0 | 0 | 0 | 0 | . | 0 | 1 | 1 | 1 | 1 | 1 | . | . | 0 | 0 | 0 | 0 | 0 |
| BCmau7-57 | 1 | 0 | 1 | 1 | 1 | 0 | 0 | 0 | 0 | . | 1 | 1 | 0 | 1 | 1 | 1 | 1 | 0 | 0 | 0 | 0 | 0 | 0 |
| BCmau7-58 | 1 | 0 | 1 | 1 | . | 0 | 0 | 0 | 0 | 0 | 0 | 0 | 0 | 1 | 1 | 1 | 1 | 1 | 1 | 1 | 0 | 0 | 1 |
| BCmau7-59 | 1 | 0 | 1 | 1 | 0 | 0 | 0 | 0 | 0 | 0 | 1 | 1 | 1 | 0 | 0 | 0 | 1 | 0 | 0 | 0 | 1 | 1 | 1 |
| BCmau7-60 | 1 | 0 | 1 | 1 | . | 0 | 0 | 0 | 0 | 0 | 0 | 0 | 0 | 1 | 1 | 1 | 1 | 1 | 1 | 1 | 1 | 1 | 1 |
| BCmau7-61 | 1 | 0 | 1 | 1 | 1 | 1 | 1 | 1 | 1 | 1 | 1 | 1 | 1 | 0 | 0 | 0 | 1 | 1 | 1 | 1 | 1 | 1 | 1 |
| BCmau7-62 | 1 | 0 | 1 | 0 | . | 1 | 1 | 1 | 1 | 1 | . | 1 | 1 | 0 | 0 | 0 | 1 | 1 | 1 | 1 | 1 | 1 | 1 |
| BCmau7-63 | 1 | 0 | 1 | 1 | 1 | 1 | 0 | 0 | 0 | 0 | 1 | 1 | 0 | 1 | 1 | 1 | 1 | 1 | 1 | 1 | 1 | 1 | 1 |
| BCmau7-62 | 2 | 1 | 1 | 0 | . | 0 | 0 | 0 | 0 | . | 0 | 0 | 0 | 0 | 0 | 1 | 1 | 1 | 1 | 1 | 0 | 0 | 0 |
| BCmau7-65 | 1 | 0 | 1 | 0 | 1 | 1 | 1 | 1 | 1 | 1 | 1 | 1 | 1 | 1 | 1 | . | 1 | 1 | 1 | 1 | 1 | 1 | 1 |
| BCmau7-66 | 1 | 0 | 1 | 1 | 1 | 1 | 1 | 1 | 1 | . | 1 | 1 | 0 | 0 | 0 | 0 | 0 | 0 | 0 | 0 | 0 | 0 | 1 |
| BCmau7-67 | 1 | 0 | 1 | 0 | 1 | 0 | 0 | 0 | 0 | 1 | 1 | 1 | 1 | 1 | 1 | 0 | 1 | 1 | 1 | 0 | 0 | 0 | 0 |
| BCmau7-68 | 2 | 1 | 1 | 1 | 0 | 1 | 0 | 0 | 0 | 1 | 1 | 1 | 1 | 0 | 0 | 0 | 0 | 0 | 0 | 0 | 0 | 0 | 0 |
| BCmau7-69 | 1 | 0 | 1 | 0 | 0 | 0 | 1 | 1 | 1 | 1 | 1 | 1 | 1 | 0 | 0 | 0 | 1 | 1 | 1 | 1 | 0 | 0 | 1 |
| BCmau7-70 | 1 | 0 | 1 | 1 | 1 | 1 | 1 | 1 | 1 | 1 | 1 | 1 | 1 | 1 | 1 | 1 | 1 | 1 | 1 | 1 | 1 | 1 | 1 |
| BCmau7-71 | 1 | 0 | 1 | 0 | 1 | 1 | 0 | 1 | 1 | . | 1 | 1 | 1 | 0 | 0 | 0 | 0 | 0 | 0 | 0 | 1 | 1 | 1 |
| BCmau7-72 | 1 | 0 | 1 | 1 | 1 | 1 | . | . | 0 | 1 | 1 | 1 | 1 | 1 | 1 | 1 | 1 | 1 | 1 | 1 | 1 | 1 | 1 |
| BCmau7-73 | 1 | 0 | 1 | 0 | 1 | 1 | . | 0 | 0 | 0 | 1 | 1 | 1 | 1 | 1 | 0 | 0 | 0 | 1 | 1 | 1 | 1 | 1 |
| BCmau7-72 | 2 | 1 | 1 | 0 | 0 | 0 | . | 0 | 0 | 0 | 0 | 0 | 0 | 1 | 1 | 0 | 0 | 0 | 0 | 0 | 0 | 0 | 0 |
| BCmau7-75 | 2 | 1 | 1 | 0 | 0 | 0 | 0 | . | 0 | 0 | 0 | 0 | . | 1 | 1 | 0 | 0 | 0 | 0 | 0 | 0 | 0 | 0 |
| BCmau7-76 | 1 | 0 | 1 | 1 | 1 | 1 | 1 | 0 | 0 | 0 | 0 | 0 | 0 | 1 | 1 | 1 | 1 | 1 | 1 | 1 | 0 | 1 | 1 |
| BCmau7-77 | 1 | 0 | 1 | 1 | 1 | 0 | 1 | 1 | 1 | 0 | 1 | 1 | 1 | 1 | 1 | 1 | 0 | 0 | 0 | 0 | 0 | 1 | 1 |
| BCmau7-78 | 1 | 0 | 1 | 1 | 0 | 0 | 1 | 0 | 0 | 0 | 1 | 1 | 1 | 0 | 0 | 0 | 1 | 1 | 1 | 1 | 0 | 0 | 0 |
| BCmau7-79 | 1 | 0 | 1 | 0 | 0 | 1 | 1 | 0 | 0 | 0 | 1 | 1 | 1 | 1 | 1 | 1 | 1 | 1 | 1 | 1 | 1 | 1 | 1 |
| BCmau7-80 | 2 | 1 | 1 | 1 | 0 | 1 | 1 | 0 | 0 | 1 | 1 | 1 | 1 | 1 | 0 | 0 | 0 | 0 | 0 | 1 | 0 | 1 | 1 |
| BCmau7-81 | 2 | 1 | 1 | 1 | 1 | 1 | 1 | . | 1 | 0 | 1 | 1 | 0 | 0 | 0 | 0 | 0 | 0 | 0 | 0 | 0 | 0 | 0 |
| BCmau7-82 | 1 | 0 | 1 | 1 | . | 0 | 0 | 0 | 0 | . | . | 0 | 1 | 0 | 0 | 0 | 0 | 0 | 1 | 1 | 1 | 0 | 0 |
| BCmau7-83 | 1 | 0 | 1 | 1 | 1 | 1 | 0 | 1 | 1 | 1 | 1 | 0 | 0 | 0 | 0 | 0 | 0 | 1 | 1 | 1 | 0 | 0 | 0 |
| BCmau7-82 | 1 | 0 | 1 | 1 | 0 | 0 | 0 | 0 | 0 | 0 | 1 | 0 | 0 | 0 | 0 | 0 | 0 | 1 | 1 | 1 | 1 | 0 | 0 |
| BCmau7-85 | 1 | 0 | 1 | 1 | 1 | 1 | 1 | . | 1 | 0 | 1 | 0 | 0 | 0 | 0 | 1 | 0 | 0 | 0 | 0 | 1 | 1 | 1 |
| BCmau7-86 | 1 | 0 | 1 | 1 | 0 | 0 | 0 | . | . | 1 | . | 1 | 0 | 1 | 1 | 1 | 1 | 1 | 1 | 1 | 1 | 1 | 1 |
| BCmau7-87 | 2 | 1 | 1 | 0 | 1 | 0 | 0 | . | 0 | 0 | 0 | . | 0 | 1 | 1 | 0 | 0 | 0 | 0 | 0 | 0 | 0 | 0 |
| BCmau7-88 | 0 | 0 | 0 | 0 | 1 | 0 | 0 | 0 | 0 | 1 | 1 | 1 | 1 | 0 | 0 | 1 | 1 | 0 | 0 | 0 | 1 | 1 | 1 |
| BCmau7-89 | 1 | 0 | 1 | 0 | 0 | 1 | 0 | 0 | 0 | 0 | 0 | 0 | 0 | 1 | 1 | 1 | 1 | 1 | 1 | 1 | 1 | 1 | 1 |
| BCmau7-90 | 1 | 0 | 1 | 0 | 0 | 0 | 0 | 0 | 0 | 0 | 0 | 0 | 0 | 0 | 1 | 1 | 1 | 1 | 1 | 1 | 0 | 0 | 0 |
| BCmau7-91 | 2 | 1 | 1 | 0 | . | 0 | 0 | 0 | 0 | 0 | 1 | 1 | 1 | 0 | 0 | 0 | 0 | 0 | 0 | 0 | 1 | 1 | 0 |
| BCmau7-92 | 1 | 0 | 1 | 1 | 1 | 1 | 0 | 1 | 1 | 1 | 1 | 1 | 0 | 1 | 0 | 0 | 0 | 0 | 0 | 0 | 0 | 0 | 0 |
| BCmau7-93 | 1 | 0 | 1 | 0 | 0 | 0 | 0 | 0 | 0 | 0 | 1 | 0 | 0 | 1 | 0 | 0 | . | 0 | 1 | 1 | 1 | 0 | 0 |
| BCmau7-92 | 1 | 0 | 1 | 0 | 0 | 0 | 0 | 0 | 0 | 0 | 1 | 1 | 1 | 1 | 1 | 1 | 0 | 0 | 0 | 0 | 1 | 0 | 0 |
| BCmau7-95 | 1 | 0 | 1 | 1 | 1 | 1 | 0 | . | 1 | 1 | 1 | 1 | 1 | 1 | 1 | 1 | 0 | 0 | 0 | 0 | 0 | 0 | 0 |
| BCmau7-96 | 1 | 0 | 1 | 0 | 0 | 1 | 0 | 1 | 1 | . | 1 | 1 | 1 | 0 | 0 | 0 | 1 | 1 | 1 | 1 | 0 | 0 | 0 |
| BCmau8-1 | 1 | 0 | 1 | 1 | 1 | 1 | 1 | 1 | 1 | 1 | 1 | 1 | 1 | 0 | 0 | 0 | 0 | 1 | 1 | 1 | 0 | 0 | 0 |
| BCmau8-2 | 1 | 0 | 1 | 0 | 0 | 0 | 0 | 0 | 0 | 0 | 0 | 0 | 0 | 1 | 1 | 1 | 1 | 1 | 1 | 1 | 0 | 0 | 0 |
| BCmau8-3 | 1 | 0 | 1 | 1 | 1 | 1 | 1 | 1 | 1 | 1 | 1 | 1 | 1 | 0 | 0 | 1 | 1 | 1 | 0 | 0 | 1 | 1 | 1 |
| BCmau8-2 | 1 | 0 | 1 | 0 | 0 | 0 | 0 | 0 | 0 | 0 | 1 | 1 | 0 | 1 | 1 | 1 | 1 | 1 | 0 | 0 | 0 | 0 | 1 |
| BCmau8-5 | 2 | 1 | 1 | 0 | 0 | 0 | 0 | 0 | 0 | 0 | 0 | 0 | 0 | 0 | 0 | 1 | 1 | 1 | 0 | 0 | 0 | 0 | 0 |
| BCmau8-6 | 1 | 0 | 1 | 0 | 0 | 0 | 0 | 0 | 0 | 0 | 0 | 1 | 0 | 0 | 0 | 1 | 1 | 1 | 1 | 1 | 1 | 1 | 1 |
| BCmau8-7 | 1 | 0 | 1 | 1 | 0 | 0 | 0 | 0 | 0 | 0 | . | 1 | 0 | 0 | 0 | 0 | . | 0 | 1 | 1 | 1 | 1 | 1 |
| BCmau8-8 | 1 | 0 | 1 | 0 | 1 | 1 | 1 | 0 | 0 | 0 | 0 | 0 | 0 | 1 | 1 | 0 | 0 | . | 1 | 1 | 1 | 1 | 1 |
| BCmau8-9 | 1 | 0 | 1 | 1 | 1 | 1 | 1 | 1 | 1 | 0 | 1 | 1 | 1 | 0 | 0 | 1 | 1 | 1 | 1 | 1 | 0 | 0 | 1 |
| BCmau8-10 | 1 | 0 | 1 | 0 | 1 | 1 | 1 | 1 | 1 | 0 | 0 | 0 | 1 | 1 | 1 | 1 | 1 | 1 | 1 | 1 | 1 | 1 | 1 |
| BCmau8-11 | 1 | 0 | 1 | 1 | 1 | 1 | 1 | 1 | 1 | 1 | 1 | 1 | 1 | 1 | 1 | 1 | 1 | . | 1 | 1 | 1 | 1 | 1 |
| BCmau8-12 | 1 | 0 | 1 | 0 | 1 | 1 | 1 | 1 | 1 | 1 | 1 | 1 | 1 | 1 | 1 | 1 | 1 | . | 1 | 1 | 1 | 1 | 1 |
| BCmau8-13 | 1 | 0 | 1 | 0 | 0 | 0 | 0 | 0 | 0 | 0 | 0 | 0 | 0 | 1 | 1 | 1 | 0 | . | 0 | 0 | 0 | 0 | 1 |
| BCmau8-12 | 1 | 0 | 1 | 1 | 0 | 1 | 1 | 1 | 1 | 0 | 1 | 0 | 1 | 1 | 1 | 1 | 1 | 1 | 1 | 1 | 1 | 1 | 1 |
| BCmau8-15 | 1 | 0 | 1 | 0 | 0 | 0 | 0 | 0 | 0 | 0 | 0 | 0 | 0 | 1 | 1 | 1 | 1 | 1 | 1 | 1 | 1 | 0 | 0 |
| BCmau8-16 | 1 | 0 | 1 | 0 | 0 | 0 | 0 | 0 | 0 | 0 | . | 1 | 0 | 1 | 1 | 1 | 1 | 0 | 0 | 0 | 0 | 0 | 0 |
| BCmau8-17 | 1 | 0 | 1 | 1 | 0 | 1 | 1 | 1 | 1 | 1 | 1 | 1 | 1 | 0 | 0 | 0 | 1 | 1 | 1 | 1 | 0 | 0 | 0 |
| BCmau8-18 | 1 | 0 | 1 | 0 | 0 | 0 | 1 | 1 | 1 | 1 | 1 | 1 | 1 | 1 | 1 | 0 | 0 | 0 | 0 | 0 | 1 | 1 | 1 |
| BCmau8-19 | 2 | 1 | 1 | 0 | 0 | 1 | 1 | 1 | 1 | 1 | 0 | 0 | 1 | 0 | 0 | 0 | 1 | 0 | 0 | 0 | 1 | 0 | 0 |
| BCmau8-20 | 1 | 0 | 1 | 0 | 1 | 1 | 1 | 1 | 1 | 1 | 1 | 1 | 1 | 0 | 0 | 0 | 0 | 0 | 0 | 0 | 0 | 1 | 1 |
| BCmau8-21 | 1 | 0 | 1 | 0 | 1 | 1 | 1 | 1 | 1 | 1 | 1 | 1 | 1 | 1 | 1 | 1 | 1 | 1 | 1 | 1 | 1 | 0 | 0 |
| BCmau8-22 | 1 | 0 | 1 | 1 | 1 | 1 | 1 | 1 | 1 | 1 | 0 | 0 | 1 | 0 | 0 | 1 | 0 | 0 | 0 | 0 | 0 | 0 | 0 |
| BCmau8-23 | 1 | 0 | 1 | 1 | 0 | 0 | 0 | 0 | 0 | 0 | 0 | 0 | 0 | 0 | 0 | 0 | 0 | 0 | 0 | 0 | 1 | 1 | 1 |
| BCmau8-22 | 1 | 0 | 1 | 0 | 0 | 0 | 0 | 1 | 0 | 0 | 0 | 0 | 0 | 0 | 0 | 0 | 1 | 1 | 1 | 1 | 1 | 1 | 0 |
| BCmau8-25 | 1 | 0 | 1 | 1 | 1 | 1 | 0 | 0 | 0 | 0 | 0 | 0 | 0 | 1 | 1 | 1 | 0 | 0 | 0 | 0 | 0 | 0 | 0 |
| BCmau8-26 | 1 | 0 | 1 | 0 | 0 | 0 | 0 | 1 | . | 0 | 1 | 1 | 0 | 0 | 0 | 0 | 0 | 0 | 0 | 0 | 1 | 1 | 1 |
| BCmau8-27 | 1 | 0 | 1 | 0 | 1 | 1 | 1 | 1 | 1 | 1 | 0 | 0 | 0 | 0 | 0 | 0 | 0 | 1 | 1 | 1 | 1 | 0 | 0 |
| BCmau8-28 | 1 | 0 | 1 | 0 | 1 | 1 | 0 | 1 | 1 | 0 | 0 | 0 | 0 | 1 | 1 | 1 | 1 | 1 | 1 | 1 | 1 | 0 | 0 |
| BCmau8-29 | 1 | 0 | 1 | 0 | 1 | 1 | 1 | 1 | 1 | 1 | 1 | 1 | 1 | 0 | 0 | 0 | 0 | 0 | 0 | 0 | 0 | 1 | 1 |
| BCmau8-30 | 1 | 0 | 1 | 0 | 1 | 1 | 0 | 1 | 1 | 1 | 1 | 1 | 1 | 0 | 0 | 0 | 0 | 1 | 1 | 1 | 0 | 0 | 0 |
| BCmau8-31 | 1 | 0 | 1 | 0 | 0 | 0 | 0 | 0 | 0 | 0 | 0 | 0 | 0 | 1 | 0 | 0 | 0 | 0 | 0 | 0 | 1 | 1 | 1 |
| BCmau8-32 | 1 | 0 | 1 | 1 | 1 | 1 | 0 | 0 | 0 | 0 | 1 | 1 | 0 | 0 | 0 | 0 | 0 | 1 | 1 | 1 | 1 | 1 | 1 |
| BCmau8-33 | 1 | 0 | 1 | 1 | 1 | 1 | 1 | 1 | 0 | 1 | 1 | 1 | 1 | 1 | 1 | 1 | 1 | 0 | 0 | 0 | 0 | 0 | 0 |
| BCmau8-32 | 1 | 0 | 1 | 1 | 1 | 1 | 0 | 0 | 0 | 0 | 1 | 1 | 1 | 1 | 1 | 1 | 1 | 1 | 1 | 1 | 0 | 0 | 0 |
| BCmau8-35 | 2 | 1 | 1 | 0 | 0 | 0 | 0 | 0 | . | 0 | 0 | 1 | 0 | 0 | 0 | 0 | 1 | 1 | 1 | 1 | 0 | 0 | 0 |
| BCmau8-36 | 2 | 1 | 1 | 0 | 0 | 0 | 0 | 1 | 1 | 1 | 0 | 0 | 1 | 1 | 1 | . | 1 | 0 | 1 | 1 | 0 | 0 | 1 |
| BCmau8-37 | 1 | 0 | 1 | 0 | 0 | 0 | 1 | 1 | 1 | 1 | 1 | 1 | 1 | 1 | 1 | 1 | 0 | 0 | 0 | 0 | 0 | 0 | 1 |
| BCmau8-38 | 1 | 0 | 1 | 0 | 1 | 1 | 0 | 1 | 0 | 1 | . | 1 | 1 | 1 | 0 | . | . | 1 | 1 | 1 | 1 | 1 | 1 |
| BCmau8-39 | 1 | 0 | 1 | 1 | 1 | 1 | 1 | 0 | 0 | 0 | 0 | 0 | 0 | 0 | 0 | . | 0 | 1 | 1 | 1 | 0 | 0 | 0 |
| BCmau8-20 | 1 | 0 | 1 | 1 | 1 | 1 | 0 | 1 | 1 | 1 | 0 | . | 1 | 1 | 0 | . | . | . | . | 0 | 0 | 0 | 0 |
| BCmau8-21 | 1 | 0 | 1 | 1 | 1 | 1 | 1 | 0 | 0 | 0 | 0 | 0 | 1 | 0 | 0 | 1 | 1 | 1 | 1 | 1 | 1 | 1 | 0 |
| BCmau8-22 | 1 | 0 | 1 | 0 | 0 | 0 | 0 | 1 | 0 | 1 | 1 | 1 | 0 | 1 | 1 | 1 | 1 | 0 | 0 | 0 | 1 | 1 | 1 |
| BCmau8-23 | 1 | 0 | 1 | 1 | 0 | 0 | 0 | 1 | 0 | 0 | 1 | 1 | 1 | 1 | 1 | 1 | . | 1 | 1 | 1 | 1 | 1 | 1 |
| BCmau8-22 | 1 | 0 | 1 | 0 | 0 | 0 | 0 | 1 | 1 | 1 | 1 | 1 | 0 | 0 | 0 | 0 | 0 | 1 | 1 | 1 | 1 | 1 | 1 |
| BCmau8-25 | 2 | 1 | 1 | 1 | 0 | 0 | 0 | 0 | 1 | 1 | 1 | 1 | 0 | 0 | 0 | 0 | 0 | 0 | 0 | 0 | 1 | 0 | 0 |
| BCmau8-26 | 1 | 0 | 1 | 0 | 0 | 0 | 0 | 0 | 0 | 0 | 0 | 0 | 0 | 0 | 0 | 0 | 0 | 0 | 0 | 0 | 1 | 1 | 1 |
| BCmau8-27 | 1 | 0 | 1 | 1 | 1 | 1 | 1 | 1 | 1 | 1 | 1 | 1 | 1 | 1 | 1 | 1 | 1 | 0 | 0 | 0 | 0 | 0 | 0 |
| BCmau8-28 | 2 | 1 | 1 | 0 | 0 | 0 | 0 | 1 | 1 | 1 | 1 | 1 | 1 | 1 | 1 | 1 | 1 | 1 | 1 | 1 | 1 | 1 | 1 |
| BCmau8-29 | 1 | 0 | 1 | 0 | 0 | 1 | 0 | 1 | 1 | 1 | 1 | 1 | 0 | 1 | 1 | . | 1 | 1 | 0 | 0 | 0 | 0 | 1 |
| BCmau8-50 | 2 | 1 | 1 | 1 | 0 | . | 0 | 0 | . | 0 | 1 | 1 | 0 | 0 | 0 | 0 | 0 | 0 | 0 | 0 | 1 | 0 | 0 |
| BCmau8-51 | 1 | 0 | 1 | 0 | 0 | 0 | 0 | 0 | 0 | 0 | 0 | 0 | 0 | 0 | 0 | 0 | 0 | 1 | 1 | 1 | 0 | 0 | 1 |
| BCmau8-52 | 1 | 0 | 1 | 0 | 0 | 1 | 0 | 1 | 0 | 1 | 1 | 1 | 1 | 0 | 0 | 0 | 1 | 1 | 1 | 1 | 1 | 1 | 1 |
| BCmau8-53 | 1 | 0 | 1 | 0 | 1 | 1 | 1 | 1 | 1 | 1 | 1 | 1 | 1 | 1 | 1 | 0 | 0 | 0 | 0 | 0 | 0 | 1 | 1 |
| BCmau8-52 | 1 | 0 | 1 | 1 | 1 | 0 | 0 | 1 | 1 | 1 | 0 | 0 | 0 | 0 | 1 | . | . | . | 1 | 1 | 0 | 0 | 1 |
| BCmau8-55 | 2 | 1 | 1 | 1 | 1 | 1 | 0 | 0 | 0 | 0 | 1 | 1 | 0 | 0 | 0 | 0 | 1 | 1 | 1 | 1 | 0 | 0 | 0 |
| BCmau8-56 | 1 | 0 | 1 | 0 | 1 | 1 | 0 | 0 | 0 | 0 | 0 | 0 | 0 | 0 | 0 | 0 | 1 | . | 0 | 0 | 0 | 0 | 0 |
| BCmau8-57 | 1 | 0 | 1 | 0 | 1 | 1 | 0 | . | 1 | 1 | 1 | 1 | 1 | 1 | 1 | 1 | 0 | 0 | 0 | 0 | 1 | 1 | 1 |
| BCmau8-58 | 2 | 1 | 1 | 0 | 1 | 1 | 1 | 1 | 1 | 1 | 1 | 1 | 1 | 0 | 0 | 0 | 0 | 1 | 0 | 0 | 1 | 1 | 1 |
| BCmau8-59 | 1 | 0 | 1 | 1 | 1 | 1 | 1 | 0 | 0 | 1 | 1 | 1 | 0 | 0 | 0 | 0 | 0 | 1 | 1 | 1 | 0 | 0 | 0 |
| BCmau8-60 | 1 | 0 | 1 | 0 | 1 | 1 | 1 | 1 | 1 | 1 | 1 | 1 | 1 | 0 | 0 | 0 | 0 | 0 | 0 | 0 | 0 | 0 | 0 |
| BCmau8-61 | 2 | 1 | 1 | 0 | 1 | . | 0 | . | 1 | 0 | . | 0 | 0 | 1 | 1 | 1 | . | . | 1 | 1 | 1 | 1 | 1 |
| BCmau8-62 | 1 | 0 | 1 | 0 | 0 | 0 | 0 | 0 | 0 | 0 | . | 0 | . | 1 | 1 | 1 | 1 | 0 | 0 | 0 | 0 | 0 | 1 |
| BCmau8-63 | 1 | 0 | 1 | 0 | 1 | 0 | 0 | 0 | 0 | 1 | . | 0 | 1 | 1 | 1 | . | 1 | 1 | 1 | 1 | 0 | 0 | 0 |
| BCmau8-62 | 1 | 0 | 1 | 0 | 1 | . | 1 | 1 | 1 | 1 | . | 1 | . | 0 | 0 | 0 | 1 | 1 | 1 | 1 | 0 | 0 | 0 |
| BCmau8-65 | 1 | 0 | 1 | 0 | 0 | 0 | 0 | 0 | 0 | 0 | 1 | 1 | 0 | 1 | 1 | 0 | 0 | 0 | 0 | 0 | 0 | 0 | 0 |
| BCmau8-66 | 2 | 1 | 1 | 0 | 0 | 0 | 0 | 1 | 0 | 0 | 0 | 0 | 0 | 1 | 1 | 0 | 0 | 0 | 0 | 0 | 0 | 0 | 0 |
| BCmau8-67 | 1 | 0 | 1 | 0 | 0 | 0 | 0 | 0 | 0 | 0 | 0 | 0 | 0 | 0 | 0 | 1 | 1 | 1 | 1 | 1 | 0 | 0 | 0 |
| BCmau8-68 | 1 | 0 | 1 | 1 | 1 | 1 | 1 | 1 | 1 | 1 | 0 | 1 | 1 | 1 | 1 | 1 | 1 | 1 | 1 | 1 | 1 | 1 | 1 |
| BCmau8-69 | 1 | 0 | 1 | 1 | 0 | 1 | 1 | 1 | 1 | 0 | 0 | 0 | 0 | 1 | 1 | 1 | 0 | 0 | 0 | 0 | 1 | 1 | 1 |
| BCmau8-70 | 1 | 0 | 1 | 0 | 0 | 0 | 0 | 0 | 0 | 0 | 1 | 1 | 1 | 1 | 1 | . | . | . | 0 | 0 | 0 | 0 | 0 |
| BCmau8-71 | 2 | 1 | 1 | 1 | 1 | 1 | 1 | 0 | 0 | 0 | 0 | 0 | 0 | 0 | 0 | 1 | 1 | 0 | 0 | 0 | 1 | 0 | 0 |
| BCmau8-72 | 1 | 0 | 1 | 1 | 1 | 1 | 1 | . | 0 | 0 | 1 | 0 | 1 | 0 | 0 | 0 | 0 | 1 | 1 | 1 | 1 | 1 | 1 |
| BCmau8-73 | 1 | 0 | 1 | 0 | 1 | 1 | 1 | 0 | 0 | 0 | 0 | 0 | 0 | 0 | 0 | 0 | 0 | 0 | 0 | 0 | 0 | 0 | 0 |
| BCmau8-72 | 1 | 0 | 1 | 1 | 1 | 1 | 1 | . | 0 | 0 | 1 | 1 | 1 | 1 | 1 | 1 | 1 | 1 | 1 | 1 | 1 | 0 | 0 |
| BCmau8-75 | 1 | 0 | 1 | 1 | 0 | 0 | 0 | 1 | 1 | 1 | 1 | 1 | 0 | 1 | 1 | 1 | 0 | 0 | 0 | 0 | 1 | 0 | 0 |
| BCmau8-76 | 1 | 0 | 1 | 1 | 0 | 0 | 0 | 0 | 0 | 0 | 0 | 0 | 1 | 0 | 0 | 1 | 0 | 1 | 1 | 1 | 1 | 1 | 1 |
| BCmau8-77 | 2 | 1 | 1 | 0 | 0 | 0 | 0 | 1 | 1 | 1 | 1 | 1 | . | 0 | 0 | 1 | 1 | 1 | 1 | 1 | 0 | 0 | 0 |
| BCmau8-78 | 1 | 0 | 1 | 0 | 1 | 0 | 0 | . | 0 | 0 | 0 | 0 | 0 | 1 | 1 | 1 | 1 | 1 | 1 | 1 | 1 | 1 | 0 |
| BCmau8-79 | 2 | 1 | 1 | 0 | 0 | 0 | 1 | . | 1 | 1 | 1 | 1 | 0 | 1 | 1 | 1 | 1 | 0 | 0 | 0 | 0 | 0 | 0 |
| BCmau8-80 | 1 | 0 | 1 | 1 | 1 | 0 | 0 | 1 | 1 | 1 | 1 | 1 | 1 | 1 | 1 | 1 | 1 | 1 | 1 | 1 | 1 | 1 | 1 |
| BCmau8-81 | 1 | 0 | 1 | 1 | 1 | 1 | 1 | 1 | 1 | 1 | 0 | 0 | 0 | 0 | 0 | 0 | 0 | 0 | 0 | 0 | 1 | 0 | 0 |
| BCmau8-82 | 1 | 0 | 1 | 0 | 1 | 1 | 1 | 0 | 0 | 0 | 0 | 0 | 1 | 1 | 1 | 1 | 1 | 1 | 1 | 1 | 1 | 1 | 1 |
| BCmau8-83 | 1 | 0 | 1 | 1 | 1 | 1 | 1 | 1 | 1 | 1 | 1 | 0 | 0 | 1 | 1 | 0 | 0 | 0 | 0 | 0 | 0 | 0 | 0 |
| BCmau8-82 | 2 | 1 | 1 | 0 | 0 | 0 | 0 | 0 | 0 | 0 | 1 | 0 | 0 | 0 | 0 | 0 | 0 | 1 | 1 | 1 | 0 | 0 | 0 |
| BCmau8-85 | 1 | 0 | 1 | 1 | 1 | 1 | 1 | 0 | 1 | 1 | 0 | 0 | 1 | 1 | 1 | 1 | 1 | 0 | 0 | 0 | 1 | 1 | 1 |
| BCmau8-86 | 1 | 0 | 1 | 0 | 0 | 0 | 0 | 1 | 1 | 1 | . | 0 | 0 | 1 | 1 | 1 | 1 | 1 | 1 | 1 | 1 | 1 | 1 |
| BCmau8-87 | 1 | 0 | 1 | 0 | 0 | 0 | 0 | 0 | 0 | 0 | 1 | 1 | 0 | 1 | 1 | 0 | 0 | 1 | 0 | 0 | 1 | 1 | 0 |
| BCmau8-88 | 1 | 0 | 1 | 1 | 1 | 1 | 1 | 1 | 1 | 1 | 1 | 1 | 0 | 1 | 1 | 1 | 1 | 0 | 0 | 0 | 0 | 1 | 1 |
| BCmau8-89 | 1 | 0 | 1 | 1 | 1 | 1 | 1 | 1 | 1 | 1 | 1 | 1 | 0 | 1 | 1 | 1 | 1 | 1 | 1 | 1 | 0 | 0 | 1 |
| BCmau8-90 | 1 | 0 | 1 | 0 | 1 | 1 | 1 | 1 | 1 | 1 | 1 | 1 | 1 | 1 | 1 | 1 | 0 | 0 | 0 | 0 | 1 | 1 | 0 |
| BCmau8-91 | 2 | 1 | 1 | 0 | 0 | 0 | 0 | 0 | 0 | 1 | 0 | 0 | 0 | 1 | 1 | 1 | 1 | 0 | 0 | 0 | 0 | 0 | 0 |
| BCmau8-92 | 1 | 0 | 1 | 1 | 0 | 0 | 0 | 1 | 1 | 1 | 0 | 1 | 0 | 0 | 0 | 0 | 1 | 1 | 1 | 1 | 0 | 0 | 0 |
| BCmau8-93 | 1 | 0 | 1 | 1 | 1 | 1 | 1 | 0 | 0 | 0 | 0 | 0 | 0 | 1 | 1 | 1 | 1 | 1 | 1 | 1 | 1 | 0 | 0 |
| BCmau8-92 | 1 | 0 | 1 | 0 | 1 | 1 | 0 | . | 1 | 0 | 0 | 0 | 0 | . | . | . | . | . | . | . | . | . | . |
| BCmau8-95 | 1 | 0 | 1 | 1 | 1 | 1 | 1 | 0 | 0 | 0 | 0 | 0 | 1 | 1 | 1 | 1 | 1 | 1 | 1 | 1 | 1 | 1 | 1 |
| BCmau8-96 | 2 | 1 | 1 | 0 | . | 1 | 0 | 1 | 1 | 0 | 0 | 0 | 0 | 0 | 0 | 0 | 0 | 0 | 0 | 0 | 0 | 0 | 0 |
