# Supplementary material for: A Novel Approach Identifying Hybrid Sterility QTL on the Autosomes of Drosophila simulans and D. mauritiana
Source: PLoS One. 2013 Sep 5;8(9):e73325. doi: 10.1371/journal.pone.0073325 (PMC3764152; doi:10.1371/journal.pone.0073325)
Supplement: Table S3 — Proportion of genotypes for the marker closest to each QTL peak in the backcross D. mauritiana data set, sorted by sperm phenotype. (DOCX) [file pone.0073325.s003.docx]

**Table S3.** Proportion of genotypes for the marker closest to each QTL peak in the backcross *D. mauritiana* data set, sorted by sperm phenotype. ‘All 20’ shows the division of phenotypes for the entire data set of 20 markers. A genotype of ‘0’ is homozygous (*mau*/*mau*) and ‘1’ is heterozygous (*mau*/*sim*).

|  |  | **Fertility class** | | | |  | | | |
| --- | --- | --- | --- | --- | --- | --- | --- | --- | --- |
|  |  | **Number** | | |  | | **Proportion** | | |
| **Marker** | **Genotype** | **No sperm** | **Non-motile sperm** | **Motile sperm** |  | | **No sperm** | **Non-motile sperm** | **Motile sperm** |
| **All 20** |  | 86 | 490 | 92 |  | | 0.129 | 0.734 | 0.138 |
|  |  |  |  |  |  | |  |  |  |
| **1774** | 0 | 27 | 208 | 75 |  | | 0.087 | 0.671 | 0.242 |
|  | 1 | 48 | 241 | 12 |  | | 0.159 | 0.801 | 0.040 |
|  |  |  |  |  |  | |  |  |  |
| **Drogpad** | 0 | 23 | 211 | 66 |  | | 0.077 | 0.703 | 0.220 |
|  | 1 | 60 | 250 | 22 |  | | 0.181 | 0.753 | 0.066 |
|  |  |  |  |  |  | |  |  |  |
| **1457** | 0 | 34 | 229 | 65 |  | | 0.104 | 0.698 | 0.198 |
|  | 1 | 50 | 256 | 26 |  | | 0.151 | 0.771 | 0.078 |
|  |  |  |  |  |  | |  |  |  |
| **10365** | 0 | 23 | 212 | 61 |  | | 0.078 | 0.716 | 0.206 |
|  | 1 | 47 | 196 | 20 |  | | 0.179 | 0.745 | 0.076 |
|  |  |  |  |  |  | |  |  |  |
| **3880** | 0 | 26 | 219 | 65 |  | | 0.084 | 0.706 | 0.210 |
|  | 1 | 52 | 247 | 23 |  | | 0.161 | 0.767 | 0.071 |
|  |  |  |  |  |  | |  |  |  |
| **17066** | 0 | 31 | 216 | 72 |  | | 0.097 | 0.677 | 0.226 |
|  | 1 | 52 | 266 | 18 |  | | 0.155 | 0.792 | 0.054 |
|  |  |  |  |  |  | |  |  |  |
| **23001** | 0 | 32 | 198 | 73 |  | | 0.106 | 0.653 | 0.241 |
|  | 1 | 52 | 286 | 17 |  | | 0.146 | 0.806 | 0.048 |
